# MOFs for next-generation cancer therapeutics through a biophysical approach—a review

**DOI:** 10.3389/fbioe.2024.1397804

**Published:** 2024-06-13

**Authors:** Leon Bernet Shano, Subramani Karthikeyan, Lourdusamy John Kennedy, Shanmugavel Chinnathambi, Ganesh N. Pandian

**Affiliations:** ^1^ Department of Physics, School of Advanced Sciences, Vellore Institute of Technology (VIT), Chennai, Tamil Nadu, India; ^2^ Centre for Healthcare Advancement, Innovation and Research, Vellore Institute of Technology (VIT), Chennai, Tamil Nadu, India; ^3^ Institute for Integrated Cell-Material Sciences, Institute for Advanced Study, Kyoto University, Kyoto, Japan

**Keywords:** MOFs, drug delivery, bio-physical approach, admet, EPR

## Abstract

Metal-organic frameworks (MOFs) have emerged as promising nanocarriers for cancer treatment due to their unique properties. Featuring high porosity, extensive surface area, chemical stability, and good biocompatibility, MOFs are ideal for efficient drug delivery, targeted therapy, and controlled release. They can be designed to target specific cellular organelles to disrupt metabolic processes in cancer cells. Additionally, functionalization with enzymes mimics their catalytic activity, enhancing photodynamic therapy and overcoming apoptosis resistance in cancer cells. The controllable and regular structure of MOFs, along with their tumor microenvironment responsiveness, make them promising nanocarriers for anticancer drugs. These carriers can effectively deliver a wide range of drugs with improved bioavailability, controlled release rate, and targeted delivery efficiency compared to alternatives. In this article, we review both experimental and computational studies focusing on the interaction between MOFs and drug, explicating the release mechanisms and stability in physiological conditions. Notably, we explore the relationship between MOF structure and its ability to damage cancer cells, elucidating why MOFs are excellent candidates for bio-applicability. By understanding the problem and exploring potential solutions, this review provides insights into the future directions for harnessing the full potential of MOFs, ultimately leading to improved therapeutic outcomes in cancer treatment.

## Graphical Abstract

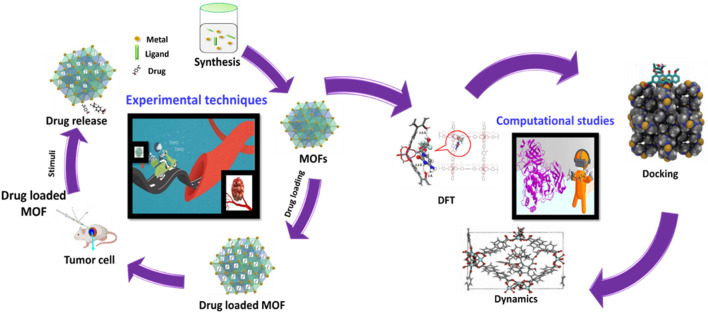

## 1 Introduction

Cancer remains one of the world’s most dreadful illnesses, which is caused by the irregular and uncontrolled division and multiplication of cells, thereby classified as benign or malignant based on their rate of growth (Siegel et al., 2019; Fatima et al., 2023). In India, one in nine individuals has a lifetime risk of developing cancer, and it will cause 9.1 lakh deaths in 2023 (https://economictimes.indiatimes.com/news/india/14-1-lakh-new-cancer-cases-9-1-lakh-deaths-in-india-who/articleshow/107352939.cms) (Sathishkumar et al., 2022). Over the past 32 years, the Global Burden of Disease (GBD) study indicates that this disease persists to be one of the most prevalent causes for mortality worldwide. It caused approximately 9.9 million deaths in 2022 globally. WHO states that the widely caused cancer count in 2022 were 2.26 million cases (breast), 2.21 million cases (lung), and 1.93 million cases (colon and rectum) (Daoui et al., 2023). Radiation exposure, age, chemicals, sun exposure, certain microbes, hereditary and lifestyle are the most prevalent risk factors for the majority of cancers (National Cancer Institute, 2017). This disorder can be treated with a various kind of modalities including surgery, radiation therapy, chemotherapy, and targeted therapy (Ibrahim et al., 2017; Karami et al., 2021). Among them, chemotherapy has been used for advanced stages of cancer, which is an untargeted treatment that causes severe side effects such as acute cholinergic gastrointestinal effects, hair loss, nausea, vomiting, cardiac problems (cardiotoxicity) and more (Kayl and Meyers, 2006). Radiation therapy resolved this issue by targeting tumor cells. To destroy these cells, it employs higher energy waves such as protons, x-rays, gamma rays, electron beams and etc., which cause the damages in tumor DNA, preventing its expansion and killing them. It may also affect healthy cells, but its effects are less severe than those of chemotherapy (Sharma et al., 2016), thus the creation of novel drug carriers is important. For improved health and prolonged human lifespan, numerous initiatives have been made to create targeted Drug Delivery Systems (DDS) that have a controlled release and enhanced therapeutic effects (Doane and Burda, 2012). Conventional DDS consists of syrups, granules, capsules, pills (oral administration), ointments, solutions or suppositories for intravenous administration. Due to the various limitations, including repeated dosing numerous times a day, pure absorption within target area, requirement of fluctuations in plasma drug level, high dose, poor bioavailability, difficult to monitor, side effects, crucial toxicities as well as premature excretion to the body, which makes these conventional DSSs incapable of achieving long-term release (Davis et al., 2008). In this regard, numerous DDSs were developed to decrease adverse effects and improve clinical efficacy (Coluccia et al., 2022).

Thus, many nanocarriers have been developed for this purpose, including polymeric micelles, liposomes, dendrimers, etc. To overcome the issues associated with low loading capacities, undesirable toxicity, and insufficient degradability, various forms of nanostructures, including nanoparticles, nanofibers, nanotubes, and nanocomposites, have been employed in drug delivery. This diversified approach aims to enhance the efficacy and safety of DDS. In this context, these innovative nanostructures can transport or carry proteins, vaccines, DNA, and enzymes (Horcajada et al., 2010). This inclusive category of nanocarriers include organic, inorganic, and hybrid nanomaterials, are used for drug delivery (Sabouni et al., 2012; Chamundeeswari et al., 2019; Karami et al., 2021).

Liposomes (Li et al., 2019a), polymeric micelles (Mousavikhamene et al., 2017), solid lipid nanoparticles (SLNs), dendrimers (Chauhan, 2018; Sherje et al., 2018), polymeric nanoparticles (PNPs) (Leong et al., 2018), and protein-based nanomaterials are examples of organic nanocarriers. These carriers provide biocompatibility and can transport a wide range of drugs. However, it has limited stability and control over drug release kinetics. Mesoporous Silica Nanoparticles (MSNs) (Kalubowilage et al., 2019; Aslam et al., 2022), graphene oxide (Jampilek and Kralova, 2021), Quantum Dots (QDs), Carbon Nano Tubes (CNTs) (Zhang et al., 2011; Cui et al., 2014) and Two-Dimensional (2D) nanomaterials such as graphene-based materials, metal nanosheets, MoS_2_, gold nanoparticles, etc., are examples of the second type of nanocarriers, which are inorganic in nature (Sharabati et al., 2022). Owing to their well-organized structure, porosity, it can release the associated drugs rather softly, although they have a lower drug loading capacity (Rezaee et al., 2022). The above said limitations in organic and inorganic nanocarriers is one of the most important fields for the new research, that is challenging to target drugs precisely and effectively.

Utilizing Metal-Organic Frameworks (MOFs) as carriers for biomedical applications is a recently explored method for overcoming these limitations. MOFs are a newly discovered category of hybrid organic-inorganic materials, achieved by the self-assembly of metals (metal chains, single metal ions or metal clusters) as well as organic linkers (Wang et al., 2009; Sun et al., 2013), whose properties are easily modifiable by altering the molecular building blocks (Li et al., 1999). In MOFs, a large number of organic linkers are covalently bound to metal ions to form a supramolecular solid material with a unique hybrid structure. It has appealed a deal of interest due to their exceptionally large surface properties, biocompatibility, flexible functionality, operationality, tunable sizes and shapes. These characteristics are extremely attractive for drug delivery (Gu and Meng, 2021). MOFs exhibit dynamic structural transformations based on flexible frameworks, leading to novel porous functions. The dynamic behavior of MOFs is a result of weak molecular interactions, such as hydrogen bonds, p-p stacking, and van der Waals forces, in addition to strong covalent and coordination bonds. These interactions allow for guest-induced structural distortion phenomena, such as crystal-to-amorphous transformation and crystal-to-crystal transformation, which can be harnessed for various applications. The stability of MOFs is crucial for their functional properties, including the ability to maintain their structure upon the removal of guest molecules from the pores and their thermal stability at high temperatures. Techniques such as X-Ray Powder Diffraction (XRPD) and Thermogravimetric (TG) measurements are commonly used to investigate the structural stability of MOFs. The microporous properties of MOFs are of great interest for applications such as gas storage, separation, and heterogeneous catalysis. The adsorption of guest molecules onto the solid surface of MOFs is governed by the pore size and shape and is influenced by interactions between the guest molecules and the surfaces. Different pore sizes lead to different adsorption behaviors, and the microporous nature of MOFs allows for the filling of molecules into nano spaces, leading to specific adsorption isotherms (Alhamami et al., 2014; Lin et al., 2020).

In 1999, William and colleagues described a copper-based metal-organic framework (Cu-MOF) utilizing benzene tricarboxylate as the linker and Cu as a metal (Chui et al., 1999). The material exhibited significant surface area, ranging from 1,000 m^2^/g to over 7,000 m^2^/g, exceptional thermal stability with a degradation temperature of up to 450°C, and large porosity, reaching up to 90% free volume high surface-to-volume ratio, it has numerous applications specially in chemistry, materials science and chemical engineering. Owing to their exceptional chemical and physical characteristics, it has been the subject of numerous investigations for a range of applications including energy areas to DDS and the result has received an increasing amount of attention.

**FIGURE 1:**
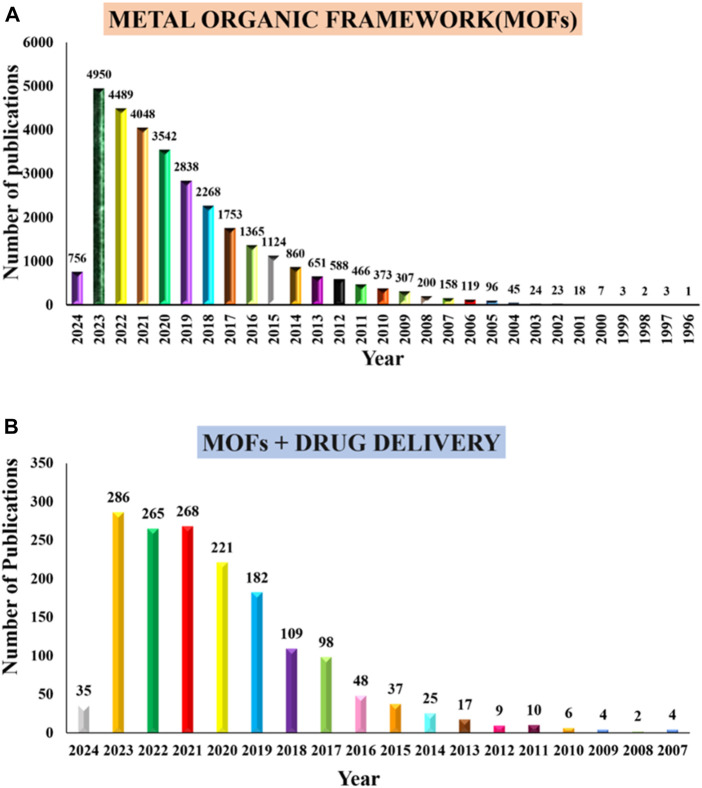
Number of publications from PubMed **(A)** “metal organic frameworks” and **(B)** “metal organic frameworks and drug delivery systems,” from 1993 to 2006, through January 2024 respectively.

Some MOFs have reached substantial loading capacity up to 81.6% ± 0.6% for various drugs (He et al., 2014). These MOF has advantages such as tunable pores, high porosity, pH sensitivity, and disadvantages such as poor encapsulation efficiency, premature drug release, and short-term circulation. To reduce these limitations, advanced MOF research, particularly on polymer and nanoparticle based, has been extensively investigated in DDS. Their large porosity, well-defined structure, flexible frameworks, wide variety of pore morphologies, extremely high surface area, comparatively low toxicity and simple chemical functionalization has contributed to making them the subject of much investigation (Sharabati et al., 2022). For the delivery of drugs both *in vitro* and vivo, nano-MOFs were studied and found to be quite efficient. As an outcome of their excellent characteristics, nano-MOFs were highly sought-after for drug delivery applications. A lot of new types of coordination polymers has emerged exponentially above the past few decades, and MOFs have become more popular than previous systems owing to their high loading capacities and biocompatibility (Lou et al., 2019). The number of publications on MOF and MOFs + drug delivery is shown in Figure 1.

Passive and active targeting are the two prevalent mechanisms for targeting anti-neoplastic agents with nanocarriers. Tumor cells are distinguished from healthy tissues by their poor lymphatic drainage and leaky blood vessels which implies that nanotherapeutics can effectively penetrate tumor tissue. Consequently, passive targeting relies on the Enhanced Permeability and Retention (EPR) effect for gathering nanocarriers at pathological spots with impaired vasculature. On the other hand, active targeting depends on particular interactions among the receptors on the pathological cell and nanocarrier, that can additionally facilitate entry of nanocarrier via receptor mediated endocytosis (Torchilin, 2010; Kotzabasaki and Froudakis, 2018). At the nanometer scale, it is possible to modify MOFs for passive targeting. This method could increase the drug concentration at the cancer site to enhance efficacy, and it can prevent drug release in normal tissues safety. MOF-based DDSs are often categorized as uncontrollable (normal), theragnostic platforms and stimuli-responsive. Here, stimuli-responsive DDSs are MOFs that react to internal or external stimuli by releasing drugs. MOFs, as drug carriers, can protect medications from degradation and deliver them precisely to the tumor site. They may also affect the cancer microenvironment, potentially increasing treatment efficacy and inhibiting drug-resistant tumor cell activity. When utilizing MOFs as nanocarriers for loading anticancer drugs or encapsulation, it is essential to incorporate specific functional molecules such as aldehyde groups or carboxyl groups (Cai et al., 2020a; Gu and Meng, 2021; Karami et al., 2021; Wang H. et al., 2022).

This comprehensive review takes readers on an exhilarating voyage through the captivating world of MOFs, exploring various aspects such as synthesis, functionalization, ADMET, EPR, encapsulation, factors influencing performance, stimuli-responsive behavior, bioimaging capabilities, photodynamic therapy, targeted drug delivery. Also, this review ensures that readers not only gain insights into the basic principles of MOFs but are also exposed to the cutting-edge applications and theoretical perspectives provided by autodocking, autodynamics, and Density Functional Theory (DFT) simulations. This all-encompassing approach makes the article particularly attractive as it serves as a one-stop reference, catering to readers across various levels of expertise and interests within the field of MOFs.

## 2 Structure and properties of MOFs

MOFs consist of two major components, namely, an inorganic metal and an organic molecule known as a linker, so it is called organic-inorganic hybrid materials. Depending on the metal and linker, the size, shape, and porosity of the MOF varies (Lawson et al., 2021). The structure of MOF can be categorized into four major levels.

**FIGURE 2:**
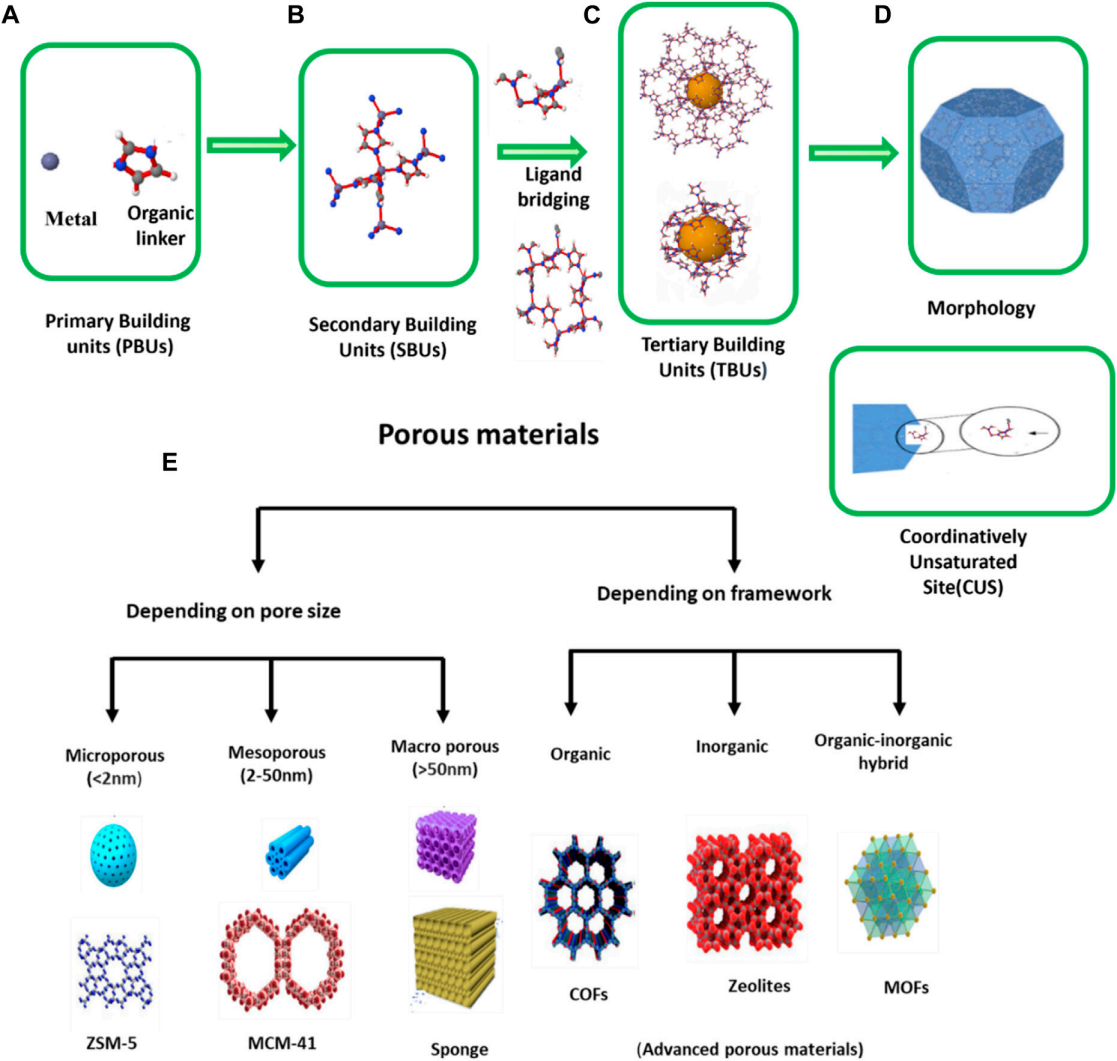
**(A)** Metal and linker, **(B)** Coordinatively Unsaturated Site (CUS) or Secondary Building Unit (SBUs), **(C)** level and inner framework structure, **(D)** morphology (Hidalgo et al., 2020). **(E)** By International Union of Pure and Applied Chemistry (IUPAC) definition (Chaudhary and Sharma, 2017) 1) microporous (e.g., Zeolite based materials (Zhang et al., 2016), pillared clays, etc.), 2) mesoporous (e.g., MCM-41 (Shutterstock), MCM-48, MCM-50, SBA-15, SBA-16, Mesoporous materials, etc.), 3) macro porous (porous gels, porous glasses, ceramic based materials, etc.) (Chaudhary and Sharma, 2017).

1) The initial level consists of metal and an organic linker which are essential components for building MOF. Strong covalent bonds connect inorganic clusters with organic multifunctional molecules (Gu and Meng, 2021). Thus, it is termed as “basic building components” of MOFs (Fatima et al., 2023).
  a) METAL: Coordination numbers and coordination geometries are the defining properties of metallic connectors. The composition of metal and, its state of oxidation, coordination quantity can vary from two to seven for different geometries, such as square-planar, linear, tetrahedral, octahedral, T or Y-shaped and pyramidal, etc., Zinc (II), Iron (III) and Zirconium (IV) are among the frequently used ions in MOFs, designed for uses in drug delivery owing to their high stability, biocompatibility, versatility and cost-effectiveness. b) LIGANDS: The majority of the organic ligands utilized in MOF synthesis, containing coordinating functional groups, such as amine, sulfonate, or nitrile, carboxylate, phosphate (Han Y. et al., 2018). Typically, the ligands utilized in MOF synthesis contain numerous amine functional groups or carboxyl which extend to a ring-based structure or an alkyl chain, such as imidazole or benzene. Integration with an ion produces a crystal-like lattice having a reiterating regularly shape. Although the majority of MOFs have rigid structures and few exhibit structural flexibility (Horcajada et al., 2010).
2) In MOFs, organic linkers are attached by metal-oxygen-carbon clusters rather than by metal ions alone. These clusters of metal-oxygen-carbon are known as “Secondary Building Units” (SBUs). It possess inherent geometric properties that facilitate MOF topology (Yaghi et al., 2003). It serves as a connecting node, coupled by linkers, resulting in the construction of the MOF network (Tranchemontagne et al., 2009). One or more aromatic rings may be present in organic SBUs, offering longer pores and larger bridges that can alter the MOF’s properties. In medical applications, nanoparticles are encased in polymer layers to generate shell-like structures with properties such as hydrophobicity. Biomolecules including peptides, proteins, amino acids, nucleobases, etc., can be used as ligands, and harmless cations such as Zn, Mg, Ca, as well as Fe, are required to produce the SBUs in Biological MOFs (bio-MOF) (Tan and Cheetham, 2011). These act as the fundamental unit cell or template for the formation of the structure of MOFs.
3) At the third level of MOF structure, known as the internal framework, multiple SBUs are connected through bridging ligands which connect the gap between two metal nodes (Mueller et al., 2006). This level of structure is important because it strongly influences the molecular and macroscopic properties of framework materials (Feng et al., 2020).
4) The exterior morphology (size, shape, and orientation) of the MOF’s final structural level is dependent on how the interior framework grows. The external morphology is altered by the method of synthesis as well as how the drug molecules were encapsulated (Liang et al., 2015; Wang L. et al., 2016). In addition, MOFs embrace Coordinatively Unsaturated Metal Sites (CUSs) which may serve as Lewis acids to facilitate loading of molecules into their surface as well as functionalize the structure (Kalmutzki et al., 2018; Lei et al., 2018; Hidalgo et al., 2020). MOFs are highly desirable for drug delivery applications owing to their excellent chemical and structural control at multiple levels. The MOF structures can be described on four distinct levels of ZIF-8, as revealed in Figures 2A–D. Porous materials are characterized by extremely large surface area, pores ranging in size from nanometers to millimeters, and low density. Typically, these materials are of natural origin such as zeolites, eggshell, rocks, sponges, and woods, while those of artificial origin include ceramics, chalk, tissue paper, bread and bricks, etc. (Kumar Pathak, 2020). Porous materials are depicted in Figure 2E according to pore size and framework.

**FIGURE 3:**
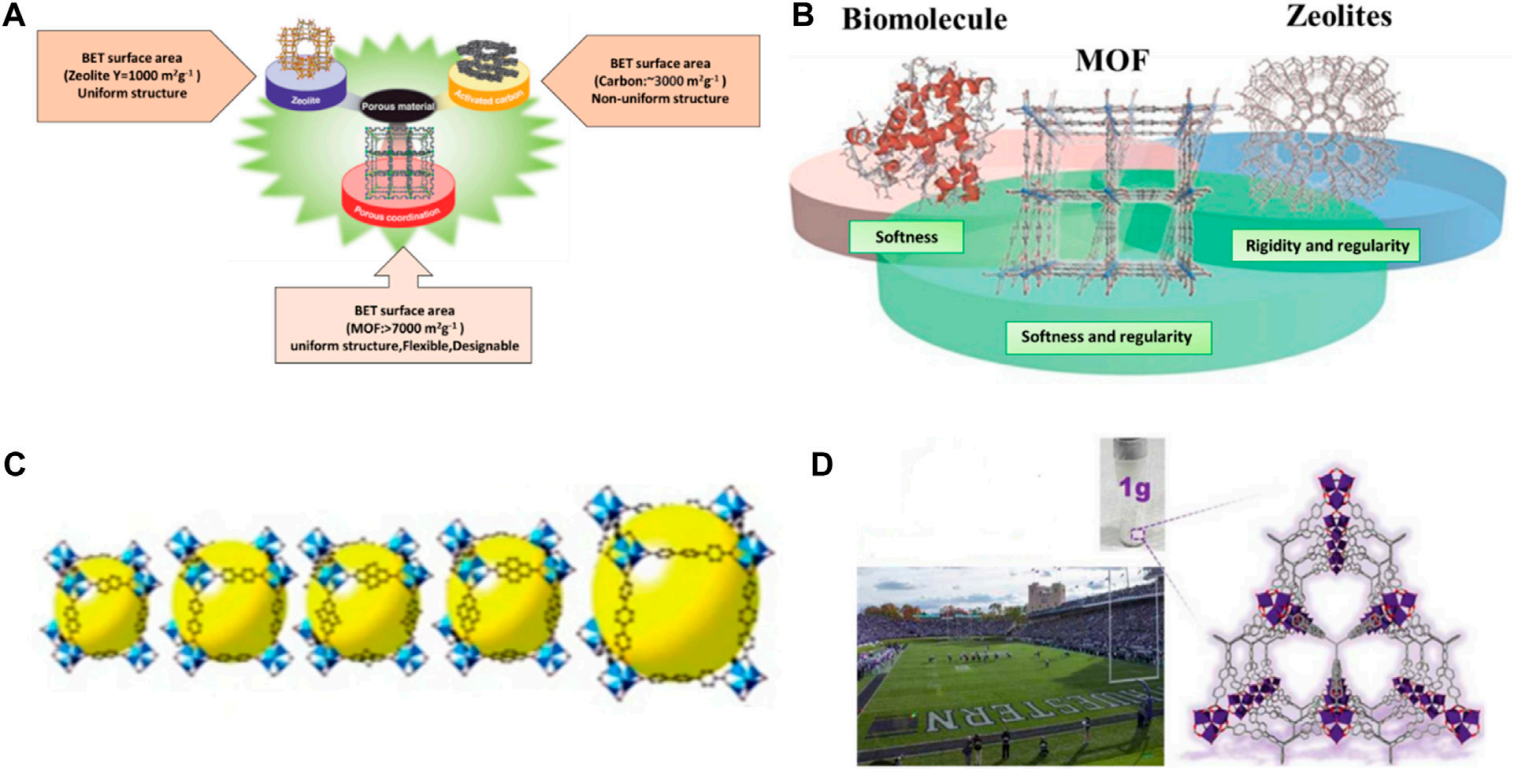
**(A)** MOF comparison to other porous materials, **(B)** MOFs exhibits softness like biomolecules and rigidity like Zeolites (Horike et al., 2009), **(C)** isoreticular synthesis of MOFs. Reprinted with permission from (Eddaoudi et al., 2002), **(D)** comparison of the MOF pore to a football stadium.

Porous materials have a larger surface area than non-porous materials of the same size and shape. This is because the internal surface area of the pores in the porous material adds to the outward surface area of the material, resulting in a greater overall surface area (Porous material). If the material has a larger surface area, there will be more interaction sites which enhance the effectiveness of the material. MOFs have a unique type of porous material which has a large surface area as well as porosity rather than conventional porous materials that include activated carbon and zeolites. Figure 3A shows the Comparison between the BET surface area of MOF, carbon and zeolites (Lin et al., 2020). Figure 3B Indicates that MOFs exhibit both softness and rigidity, like biomolecules and Zeolites, respectively. Figure 3C Isorecticular synthesis is possible which means maintaining same MOFs structure but the cavity or pore size inside can be vary (Eddaoudi et al., 2002) Figure 3D Surface area of 1 g of MOFs (NU-110) is equivalent to one football stadium. In contrast to other porous nanoparticles, MOFs are flexible to microstructural modifications by varying the type and quantity of metal ions and organic linker (Zhou et al., 2012; Mahmoodi et al., 2019c; Mahmoodi et al., 2020).

**FIGURE 4:**
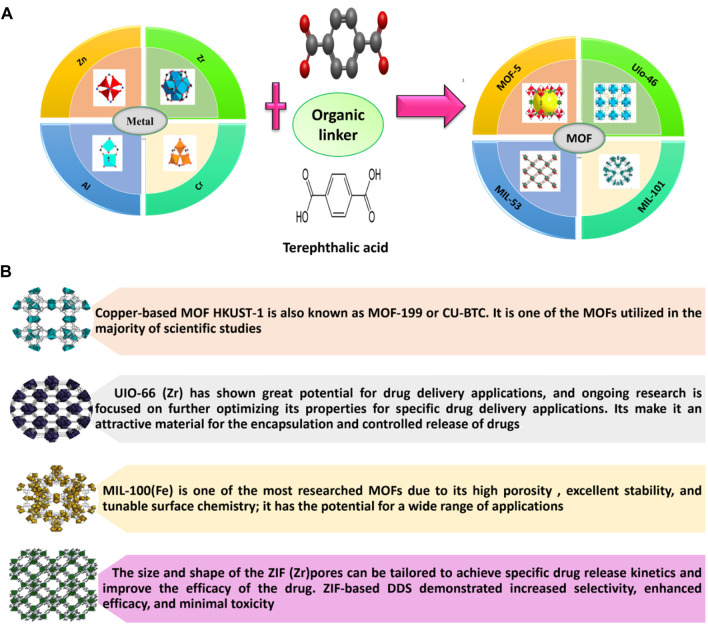
**(A)** Metal-Organic Framework (MOFs) (Perez et al., 2016; Rocío-Bautista et al., 2019). Here, Terephthalic acid adopted from (https://en.wikipedia.org/wiki/Terephthalic_acid). **(B)** Some of MOF used for drug delivery (https://www.cd-bioparticles.net/metal-organic-frameworks-mofs-materials, NovoMOF).

MOFs are designed by two architectures such as, organic molecules as bridges and metal cations as nodes (Kreno et al., 2012; Mahmoodi et al., 2019a; Mahmoodi et al., 2019b). Nanometer-sized pores can be occupied by anticancer drugs within a framework. Figure 4A indicates the combination of different metals with Terephthalic acid to produce a different type of MOF. It is composed of both inorganic clusters, including polynuclear clusters or metal ions, as well as organic polyfunctional molecules. Here, strong covalent bonds link inorganic clusters to organic multifunctional molecules. The structure of MOFs can be one-dimensional, two-dimensional, or three-dimensional (Yaghi et al., 1998; Yaghi et al., 1003; Férey, 2000). Figure 4B shows some of the most common MOFs used for drug delivery.

MOFs also exhibit additional extra characteristics for a successful drug delivery, including biocompatibility, controlled release of drugs, ease of surface modification, high chemical and thermal stability, high loading capacity, as well as tunability through a systematic approach to conjugate functional groups and/or alter the pore size (Simon-Yarza et al., 2018). Their distinctive combination of an extensive variety of pore sizes, large porosity, topologies, high surface areas, simple surface functionalization, absence of unreachable bulk volume (meso-or micro pores), and shapes (channels, cages, etc.) switchable, rigid frameworks and an infinite number of possible combinations of ligands and metals has a vast array of possible applications (Coluccia et al., 2022). NMOFs offer numerous advantages over conventional DDS, including the enumerated benefits.

1. The larger surface area (i.e., surface areas ranging between 1,000 and 10,000 m^2^/g) and porosity [up to 6 nm (Morris and Wheatley, 2008)] which enhance the drug loading capacity (Lian et al., 2017; Sun et al., 2020).
2. MOFs are biodegradable, because coordination bonds are of moderate strength. Due to the weakness of this bonds, biodegradability is crucial for controlled drug release (Anand et al., 2014; Zhang S. et al., 2020; Sun et al., 2020). They are designed to produce structures of various shapes, sizes, and chemical properties, enabling the loading of numerous drugs with distinct functionalities (Coluccia et al., 2022).
3. Tunable nature is high (Lan et al., 2011); Through an alteration in the organic linker and/or metal, it is possible to alter the porous shape structure and size, as well as its chemical properties (Sun et al., 2013).
4. Large crystallinity, that reveals distinct morphological information as well as distinct networks, has been essential when analyzing host-guest interactions (Anand et al., 2014; An et al., 2019).
5. The post-synthetic functionalization of their surfaces can increase their colloidal stability, thereby extending their time in circulation (Farha and Hupp, 2010; Tanabe and Cohen, 2011; Lu W. et al., 2014; Riccò et al., 2018; Simon-Yarza et al., 2018; Zhang S. et al., 2020).

**FIGURE 5:**
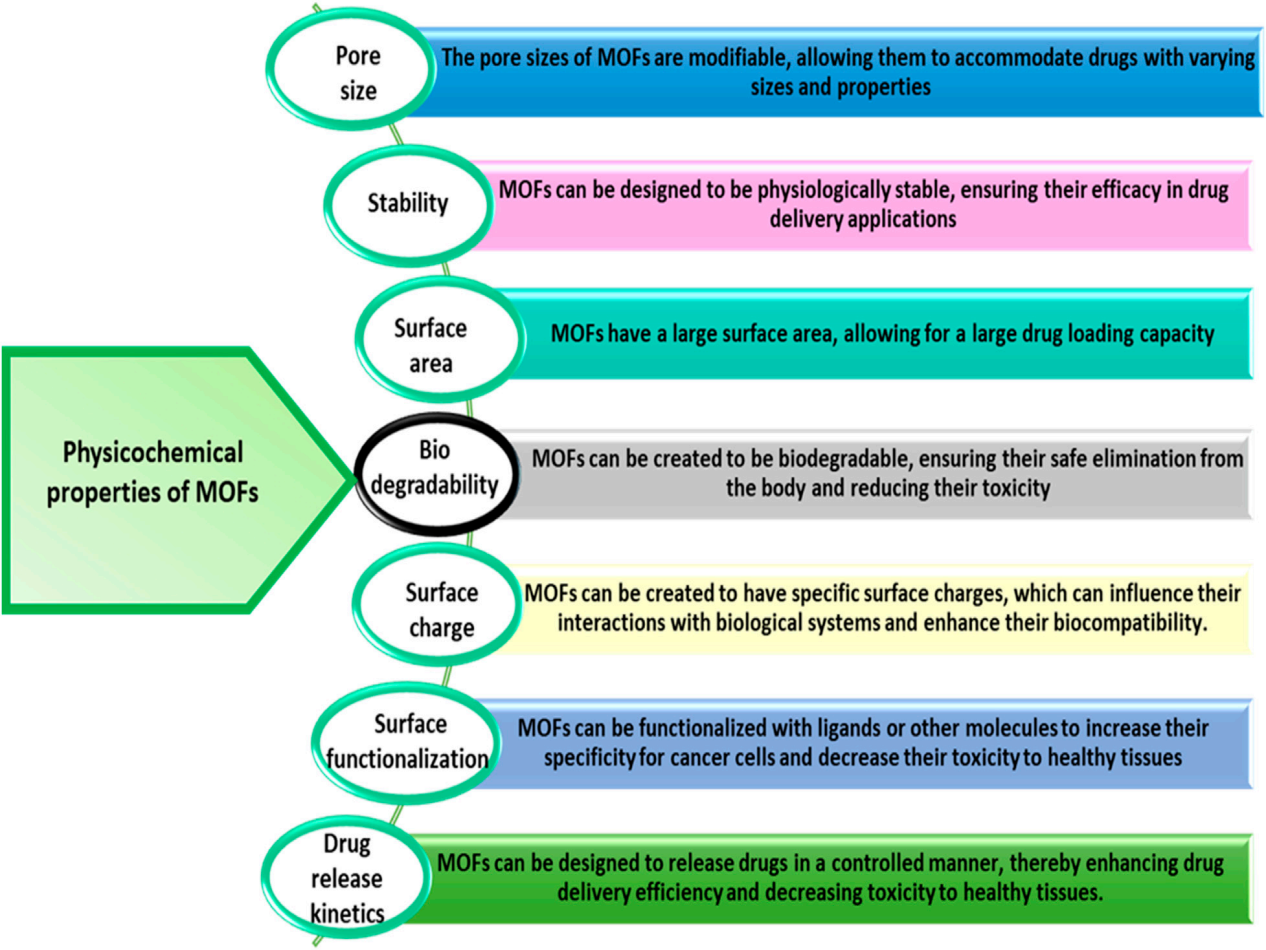
Physical and chemical properties of MOF for cancer drug delivery (Cai et al., 2020a; He et al., 2021; Mallakpour et al., 2022; Chen et al., 2023).

MOFs are among the most promising candidates to drug delivery in biomedical applications due to these exceptional properties. Until now, a series of therapeutic agents have been chosen to research into MOFs for drug delivery application. Anticancer drugs including camptothecin (Zhuang et al., 2014), doxorubicin (DOX) (Ren et al., 2014; Adhikari and Chakraborty, 2016; Chen et al., 2017; Bhattacharjee et al., 2018) cisplatin (Rieter et al., 2008), 5-fluorouracil (5-FU) (di Nunzio et al., 2014) and topotecan (Zhang F.-M. et al., 2017) are being incorporated into MOFs for cancer treatment and intracellular delivery. Now a days, researchers investigating the delivery of numerous biomolecules by MOF nanocarriers (Lu K. et al., 2014; Lismont et al., 2017). These Biomolecules are essential to biological processes and are present in living organisms (Zhuang et al., 2017). These are also macromolecules, such as nucleic acids, lipids, proteins and carbohydrates, as well as small molecules, such as fatty acids and amino acids. Biomolecular drug delivery of the molecules with crucial biological functions offers a novel approach to disease treatment (Sun et al., 2020). MOFs have shown great potential for cancer diagnosis and therapy due to their unique physicochemical and biological properties. The physicochemical properties of MOFs are designated in Figure 5. This structure allows for high surface area and tunable pore sizes, enabling efficient loading and controlled release of therapeutic agents. Many MOFs exhibit pH-responsiveness, allowing for targeted drug delivery to the acidic tumor microenvironment (Fytory et al., 2021; Yusuf et al., 2022). Additionally, it can be functionalized with targeting ligands like folic acid, lactobionic acid, or glycyrrhetinic acid to enhance selective uptake by cancer cells (Yang et al., 2023). This improves the therapeutic index and reduces off-target effects. It has also been explored as platforms for photodynamic therapy, where the framework can host photosensitizers that generate cytotoxic reactive oxygen species upon light irradiation. As an example, a recent study reported the development of a dual-ligated Zr (IV)-based nanoscale MOF (NH_2_-UiO-66) loaded with the chemotherapeutic drug doxorubicin. The MOF was decorated with both lactobionic acid and glycyrrhetinic acid to target hepatocellular carcinoma cells. This multifunctional nanoplatform demonstrated superior cytotoxicity, pH-responsive drug release, and enhanced cellular uptake compared to non-targeted or mono-ligated counterparts. Such rationally designed MOF-based DDS hold great promise for improving the efficacy of cancer therapy (Fytory et al., 2021).

The biocompatibility and toxicity of MOFs are influenced by factors such as MOF concentration, choice of metallic nodes, and organic ligands. Hydrophobicity is a crucial physicochemical characteristic affecting MOF toxicity, with increased hydrophobicity correlating to increased toxicity. Another characteristic of MOF is its biodegradability, which is influenced by the metal ion, organic ligand, and pH of the surrounding environment (Ahmadi et al., 2021). In biomedicine, enhancing the utility of MOFs requires selecting biocompatible metal centers with low toxicity. Bio-MOFs constructed from endogenous biomolecules like amino acids, peptides, nucleobases, porphyrins, amino acids, peptides, proteins, and saccharides are explored for their biocompatibility and functional diversity (Wang et al., 2020). Metals essential for human health, such as iron, zinc, and magnesium, are typically chosen for creating biocompatible MOFs (Yuan et al., 2018). Based on the metal’s lethal and daily doses, the most suitable cations are chosen to create biocompatible MOFs. For example, the following metals are suitable for building biocompatible MOFs: Ca, Mg, Zn, Fe, Ti, and Zr (Horcajada et al., 2012; Singh et al., 2021). Notably, metals like Cu (HKUST-1), Zr (UiO-67 and UiO-66), Zn (ZIF-8, MOF-74), and Fe (MIL-88, MOF-74, MIL-101, and MIL-100) and their respective families of MOF structures have been extensively explored in biomedical research, particularly for cancer, microbial infections, and inflammatory diseases (Wang et al., 2023). Therefore, the biocompatibility, potential for diverse functionality, and low toxicity of metals are taken into consideration when selecting metals for MOFs in biomedical applications. Nucleobases, especially adenine and guanine, serve as important biological ligands due to their strong coordination behavior and multiple coordination sites. Amino acids, peptides, proteins, and porphyrins, as endogenous biomolecules, contribute to both biocompatibility and versatile functionality (Wang et al., 2020). These ligands are biocompatible and have low toxicity, making them desirable for use in biomedical applications. Also carboxylates, phenolates, sulfonates, and amines are also commonly used as organic linkers to synthesize MOFs (Sharabati et al., 2022). Saccharides, representing carbohydrates, further enhance biocompatibility and functional diversity when employed as organic ligands within bio-MOFs. The coordination of these organic ligands with diverse metal centers results in the structural diversity of bio-MOFs, making them valuable for various biomedical applications (Wang et al., 2020). For instance, Bovine Serum Albumin (BSA) is often employed as a stabilizer as well as skeleton for bio-MOFs (Han L. et al., 2018).

## 3 Synthesis of MOFs

The synthesis of MOFs directly influences the crystallization of the structure, it is crucial to select a synthesis technique that can effectively regulate the physiochemical properties, including crystallinity, porosity, and morphology, of the resulting molecules (Lee et al., 2013). Moreover, environmental and economic factors have to be considered, particularly in large-scale synthesis. Based on the resulting features and frameworks, numerous different synthetic techniques can be used to build MOFs. Most common synthesis methods used in MOFs are steam-assisted conventional, mechanochemical (Pichon et al., 2006; Masoomi et al., 2015), hydrothermal (solvothermal) (Qiu and Zhu, 2009; Shen et al., 2013), non-solvothermal (Butova et al., 2016), microwave assisted (Sabouni et al., 2012; Phang et al., 2014; Babu et al., 2016), slow diffusion (Chen et al., 2005; Wang et al., 2010), green and time-saving (Mao et al., 2019), electrochemical techniques (Chen et al., 2005; Mueller et al., 2006; Campagnol et al., 2014) and sono chemical conventional. Table 1 indicates the Different synthetic routes for the production of MOFs. The preparation of MOFs involves the linkage of metal ions or clusters as the node and organic ligands as the strut, resulting in an extended infinite one-, two-, or three-dimensional network (Liu et al., 2016).

**TABLE 1:** Different synthetic routes for the production of MOFs (Stock and Biswas, 2012; Karami et al., 2021; Yusuf et al., 2022).

Synthesis	Characteristics	Description	Reaction time	Temperature	Advantage	Disadvantage
Sonochemical method	Ultrasonic radiation synthesis technique (20 kHz–10 MHz)	High-energy ultrasound waves cause the bubbles to collapse, resulting in higher pressures and temperatures that accelerate the formation, reaction and cavitation of MOFs, and their rapid production	30–120 min	25°C–50°C	• Efficient reduction in particle size • Suspensions in concentration • Produce homogeneous nucleation • Simple and eco-friendly, fast synthesis • Excellent mono dispersity • Modulate crystallization time	• Limited temperature range • May result in low crystallinity • Single crystallization is difficult • The crystals were broken by additional sonication • Poor yield
Non solvothermal synthesis	Elevated temperature and pressure, an autogenous reaction environment, controlled crystal growth, limited scalability and high purity	Typically, non-solvothermal methods for synthesizing MOFs include the reaction of organic ligands and solid metal precursors at high temperatures, with or without the help of mechanical energy or other external stimuli, to form the desired product	—	50°C–100°C	• Simplify chemical requirements • Under higher pressures	• Low yield • Large particle size • Long response time
Hydrothermal	Crystallization of MOFs in a sealed container at autogenous high pressure and the boiling point of the solvent	Organic ligands, metal salts and the solvent are combined in a sealed container at elevated pressure and temperature below critical conditions	24–96 h	50°C–180°C	• Single-step synthesis • Single crystallinity • Beneficial for crystal growth • Simple industrial conversion • Excellent mono dispersity and yield • Morphology and size control • High porosity and yields • Moderate temperature • Single and Excellent crystallinity • More productivity, smaller and more uniform crystals than non-solvothermal synthesis	• The purchasing of pressure-sealed metal tanks and heating ovens is costly • Simple to produce by-product • Needed extra solvents • Consuming a lot of energy and time • Needs specialized equipment involving sealed containers or autoclaves
Electrochemical Method	Using electrical energy for the crystallization	The metal is used as an electrode, which upon application of a voltage or current, interacting using an organic linker that dissolves with electrolyte solution together to conducting salt	10–60 min	Room temperature	• Increase the solids content • Continuous process • Mild reaction state • Less time consuming	• Needs special apparatus • Particular device • Required N_2_ environment • Lower output
Microwave assisted	The process caused by the excitation of molecules by microwave electromagnetic radiation	Microwave radiation is utilized to heat the mixture of reactants and solvents	5 min–4 h	30°C–150°C	• Narrow particle size distribution • A reduction in crystallization time and an improvement in yield • Rapid synthesis that is environmentally friendly • Easy and energy conserving • High synthesis efficiency • Use a high-frequency electric field to heat or cool the environment with electrical charges • Homogeneous morphology • Highest purity/particular phase • Shortened reaction time • Particle size controllability	• Expensive equipment • No simple and rapid industrial application • Poor production • Large single crystals are difficult to separate • Particular device • Industrial preparation is difficult
Mechanochemical	A technique for solvent-free technique involving hand grinding or ball milling	A solvent-free process in which a mechanical force, (grinding) triggers a chemical process which results in the formation of MOF crystals	30–180 min	Room temperature	• Only mechanical forces are applied • Clean energy • Room temperature • Solvent-free synthetic procedure • Environmental synthesis and eco-friendly • High yields, output and low-cost manufacturing • Further temperature and pressure are not necessary • Green method • Requiring less time	• Materials and energy consumption • Decreased pore volume • There is a secondary phase present • Restricted to particular MOFs may result in poor crystallinity • Possible structural modification • Limited crystalline
Template-assisted method	Adding templates with precursors yields porous materials	Using pre-existing materials or templates to guide the growth of MOF crystals, resulting in controlled properties such as morphology and size	6–48 h	50°C–180°C	• The synthesis of hierarchically porous MOFs • Unique MOFs having uncommon topologies or architectures	• There may be residual templating agents in the MOFs

### 3.1 Steam-assisted convention method

The steam-assisted approach eliminates the use of hazardous gases, such as HF, and has more capacity and efficiency than the standard hydrothermal method**.** This method involves converting MOF precursors or sols into crystalline porous coordination compounds**.** Developed a unique synthesis technique that can produce MIL-100 (Cr) via heat conversion without the use of HF, with a shorter reaction time of 9 h, an exceptional yield of 96%, and a greater selectivity of N_2_ over CH_4_. This approach is beneficial and well-suited for large-scale, long-term production of MIL-100 (Cr) (Wang C. et al., 2019).

### 3.2 Sonochemical method

Lately, the sono chemical method is being utilized for the fast production of MOFs because it reduces the duration required for ultra radiation-induced crystallization. In this technique, the MOF is synthesized using up to 20 kHz to 10 MHz (cyclic mechanical vibration) (Bang and Suslick, 2010). A combination of the metal salt and organic linker is added to Pyrex reactor which has a variable power output and sonicator bar without the use of external cooling (Lee et al., 2013). Ultrasound is the primary factor in cavitation’s effect on a liquid which refers to the collapse of bubbles produced by sonication in a solution and formation. It exhibits extremely proper crystallites at approximately pressures of 1,000 bar (Qiu et al., 2008). Ahn’s, et al., presented a sonochemical approach for fabricating Mg-MOF-74 nanoparticles within a 1-h timeframe following the introduction of triethylamine as a deprotonating agent. This technique shows the application of ultrasound as a facilitator in the synthesis of MOF crystals, emphasizing the benefits of sonochemical methods in MOF preparation (Hu et al., 2021). In addition, it is eco-friendly, user-friendly, applicable at room temperature, and has a significantly shorter synthesis time than other conventional synthesis processes (Xu et al., 2013; Saeed et al., 2020; Karami et al., 2021). One example of the sonochemical method is the synthesis of UiO-66-NH_2_, a zirconium-based MOF, using ZrC_l4_, 2-aminoterephthalic acid, and N, N-dimethylformamide. Sono chemically synthesized UiO-66-NH_2_ MOF exhibits a higher surface area and smaller particle sizes, resulting in an elevated adsorption capacity for CO_2_, even under conditions of low pressure (Kazemi et al., 2023).

### 3.3 Mechanochemical method

Mechanochemical reactions depend on reagents, often solids, directly absorbing mechanical energy during grinding or milling, for example, ball milling (Lee et al., 2013; Saeed et al., 2020). According to this technique, the sources of energy essential to beginning chemical processes include collisions and friction between reactants and balls. A large ball collision is necessary for a chemical reaction to occur, otherwise, just elastic deformations appear. The reaction happens rapidly (10–60 min) at ambient temperature, resulting in excellent yield (Garay et al., 2007; Kaupp, 2009). Insoluble metal oxides can be used as metal precursors alternatives to salts because they are most environmentally friendly, safer, and provide opportunities for the synthesis of novel materials (Kaupp, 2009; Karami et al., 2021).

### 3.4 Hydrothermal

Hydrothermal synthesis is a process of preparing substances by dissolving and recrystallizing powders in a sealed, pressurized container containing a solution of water (Lee et al., 2013). This method based on solvent interaction of organic ligands with metal salts and crystallization in an enclosed chamber (sealed container or autoclave), where pressure (above or at a solvent’s boiling point) and high temperature facilitate crystal growth and self-assembly. Wei Cheng et al., investigated a bimetallic MOF produced through a hydrothermal method, designated as Cd/Zr-MOF, utilizing Zr^4+^ and Cd^2+^ ions. Moreover, they examined a MOF-based Co_3_O_4_/SnO_2_ composite for ethanol detection, demonstrating its superior sensing performance over SnO_2_ nanoparticles derived from MOF (Cheng et al., 2021). Additionally, MIL-53, known for its high specific surface area and pore volume, exhibits remarkable pollutant removal capabilities (Li et al., 2022). Furthermore, the hydrothermal synthesis of HF-Free MIL-100 (Fe) has shown promise in drug delivery applications, particularly for the anti-tuberculosis drug isoniazid (INH). This MOF, synthesized without hydrofluoric acid, boasts a porous structure conducive to drug loading and release, positioning it as a potential DDS (Simon et al., 2019).

### 3.5 Solvothermal

In MOFs synthesis, the solvothermal method remains to be the most popular of the numerous synthetic techniques shown to date due to its ability to produce uniform MOF particles with high crystallinity, phase purity, and small particle sizes (Denisov et al., 2019). Solvothermal synthesis involves the reaction of metal ions and organic ligands in a solvent at temperatures above the solvent’s boiling point, enabling reactions that would not occur under standard conditions and leading to the formation of new compounds or polymorphs. Khaliesah kamal et al., focused on optimizing washing processes in the solvothermal synthesis of nickel-based MOF-74, a material with promising applications in drug delivery. Their study proposed enhancements in washing techniques, incorporating centrifugal separations after reaction and product washing steps. Through these optimizations, the study achieved a final sample demonstrating improved gas adsorption performance, with a CO_2_ uptake of 5.80 mmol/g, competitive with literature data and notably higher than samples from basic synthesis routes (Kamal et al., 2020). Additionally, the Sr/PTA MOF was synthesized using a solvothermal method, involving the reaction of strontium nitrate and 1,3,5-benzenetricarboxylic acid (BTC) in a solvent mixture of water and ethanol at 120°C for 24 h. This resulting MOF exhibited high surface area, large pore volume, and a pore size of approximately 1.2 nm, rendering it suitable for drug loading and release. Subsequently, ketoprofen-loaded Sr/PTA MOF was prepared by immersing the MOF in a ketoprofen solution for 24 h, achieving a drug loading efficiency of 48.4%. Ketoprofen release from the MOF was found to be pH-dependent, with faster release rates at lower pH values, indicating potential for targeted drug delivery in acidic environments such as inflamed joints in osteoarthritis (OA) (Li Z. et al., 2019). Furthermore, Hao Liu et al., investigated the synergistic effects of anticancer drugs delivered via ZIFs, employing solvothermal synthesis to fabricate the ZIF-8/TBHPC composite. This process involved the reaction of ZIF-8 with TBHPC (a specific anticancer drug) in a solvent under controlled conditions. The successful execution of the solvothermal synthesis procedure resulted in the formation of the ZIF-8/TBHPC composite, representing a significant advancement in the development of a potential drug delivery system aimed at enhancing cancer therapy (Liu et al., 2023).

### 3.6 Electrochemical method

**FIGURE 6:**
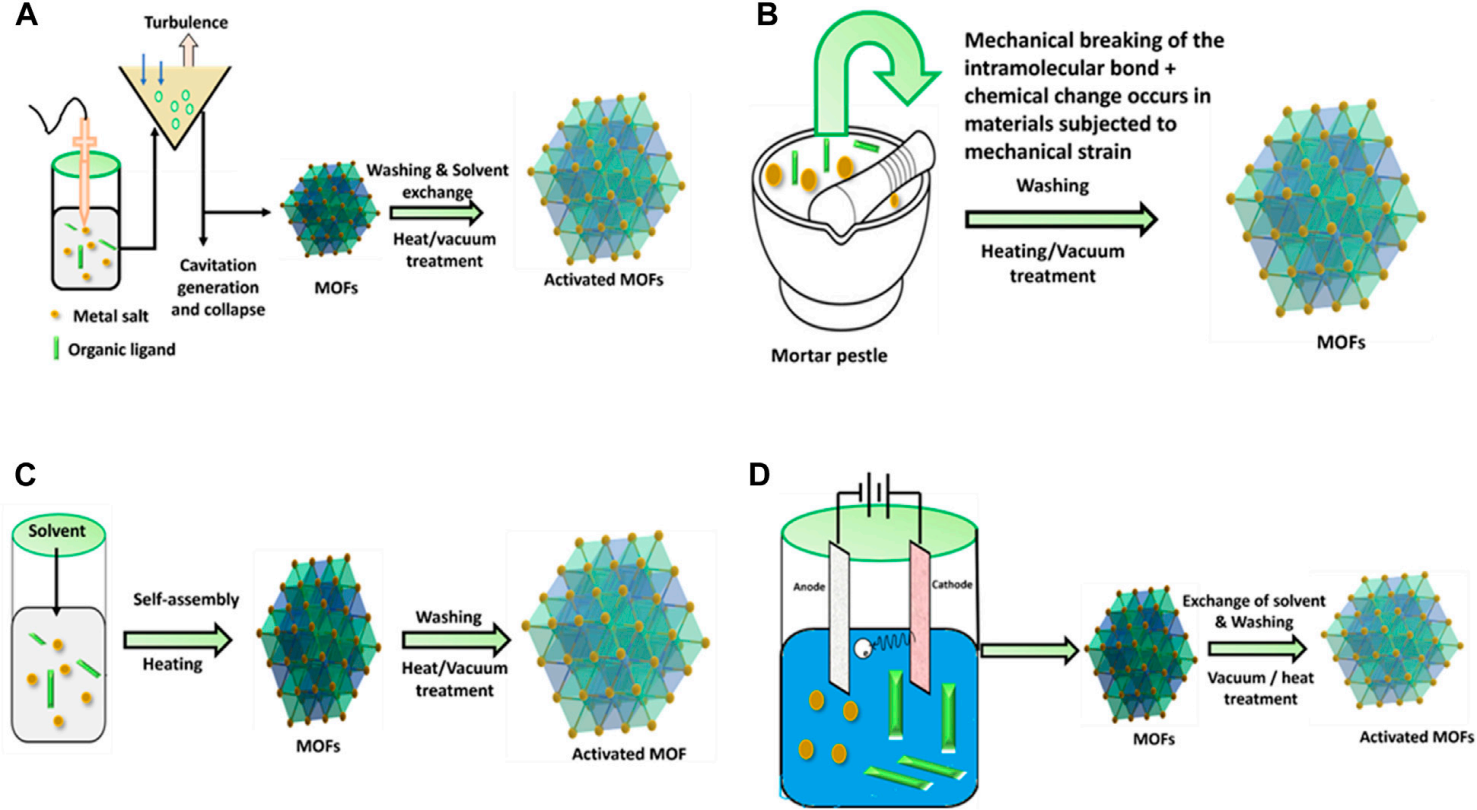
Schematic diagram of MOFs synthesis technique. **(A)** Sono chemical, **(B)** mechanochemical, **(C)** hydrothermal, **(D)** electrochemical methods.

BASF research group investigated electrochemical synthesis for the first time in 2005 for the synthesis of HKUST-1. The primary objective of reducing the concentration of anions during synthesis is to enable large-scale synthesis through anodic dissolution. Protic solvents prevent metal accumulation on the cathode, but H_2_ is generated in this process. As an alternative to these solvents, some compounds including maleic esters, acrylic, or acrylonitrile can also be utilized. The possibility of continuous operation is an additional benefit of the electrochemical route for continuous processes. Additionally, greater solids content is obtained compared to conventional batch reactions (Stock and Biswas, 2012; Khan and Shahid, 2022). This method has significant limitations as only MOF particles containing the same component metal ions as the substrate can adhere to it be manufactured (Wang A. et al., 2016). Figure 6 illustrates schematic diagram of some typical MOFs synthesis techniques.

Characterization techniques for MOFs in drug delivery have advanced over time. Some of the techniques used in this were X-Ray Diffraction (XRD), Scanning Electron Microscopy (SEM), Transmission Electron Microscopy (TEM), Fourier Transform Infrared Spectroscopy (FT-IR) and Nuclear Magnetic Resonance (NMR) spectroscopy. XRD is used to determine the crystalline structure of MOFs, while SEM and TEM provide details about their morphology. FT-IR provides valuable information about the functional groups and chemical bonds present in MOFs. NMR spectroscopy is employed to study the interaction between MOFs and drug molecules. These techniques have evolved to allow for the precise characterization of MOFs and their application in drug delivery systems (Yang et al., 2016a; Lei et al., 2018; Zong et al., 2022).

The synthesis and application of MOFs face several bottlenecks that hinder their widespread use. One significant challenge lies in the complexity of synthetic modifications required for MOFs, which can impede large-scale production and industrial use. The synthesis process involves selecting metal ions or clusters and bonding them with organic linkers to create structures in one or multiple dimensions. Various synthesis methods such as solvothermal, microwave, and slow evaporation exist, each with their advantages and limitations. For example, the solvothermal method offers a variety of morphologies but requires organic solvents or solvent mixtures. Conversely the slow evaporation method, although time-consuming, allows for MOF preparation without external energy supply. The microwave method, while faster, may require specific conditions for desired morphology and properties (Remya and Kurian, 2019). MOFs’ poor stability in water, as well as mass transfer restrictions, are additional bottlenecks preventing industrial production (Aggarwal et al., 2022). MOFs exhibit lower chemical, thermal, and hydrothermal stability compared to oxides, making them less suitable for harsh environments. The narrow parameter range for MOF synthesis further limits their versatility and scalability, complicating customization for specific applications. Moreover, the high cost of commercially available MOFs presents a barrier to their extensive adoption. Efforts are being made to explore cost-effective synthesis methods, such as utilizing waste materials, aim to address this challenge and enhance the accessibility of these promising porous nanomaterials (Naser et al., 2023).

## 4 Modification of MOFs-Cargo loading strategies

**FIGURE 7:**
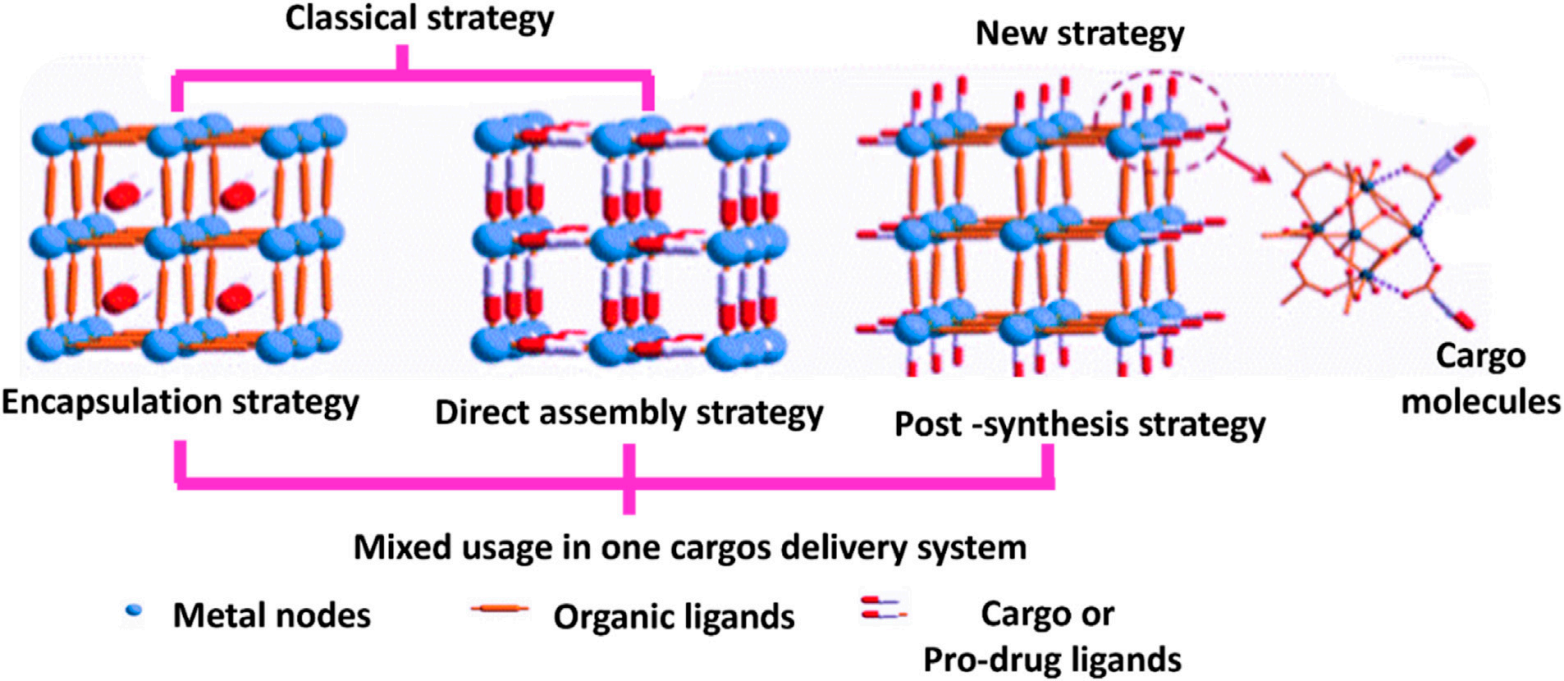
Methods for drug loading in MOFs (Wang et al., 2018).

MOF has been shown to be a stable and safe platform for the development of extremely effective DDS for cancer therapy (Lei et al., 2018; Li Y. et al., 2020). Encapsulation within MOFs has no limits to drug delivery; it is also being considered for use in the therapy of various types of cancer (Hartlieb et al., 2017) which include breast, gastric, and colon (Le et al., 2022). The drug delivery mechanism of MOFs allows for a manageable and slow release of drugs, which is a significant advantage over other DDS. The encapsulation technique involves placing cargo within MOFs (Maranescu and Visa, 2022). It requires incorporating drug molecules within the pores of MOFs to prevent their degradation as well as regulate their release. The drug can be slowly released over time, resulting in sustained therapeutic effects. In addition, encapsulation can protect the drug from enzymatic degradation and immune system clearance, allowing greater drug concentrations at the site of action. Additionally, it exhibit unique properties which includes subnetwork displacements, swelling, linker rotation and breathing which are essential for release management and drug loading (Wang A. et al., 2016). Also, there are numerous methods to connect a drug to MOF, that could indicate a medication, an enzyme, a protein, a gene, or any other therapeutically important component. MOFs have the distinctive qualities of a highly organized structure and a huge surface area. Due to this property medicines are sometimes implanted in the outside surface or enclosed in inter pores using various loading procedures (Munawar et al., 2023). For loading MOFs with huge quantities of drugs, Wang and colleagues describe three following cargo loading methods which are encapsulation, direct assembly, and post-synthesis method indicated in Figure 7.

### 4.1 Encapsulation method

Cargoes are placed in the pores or channels of MOF through noncovalent interactions, without altering the framework structures (Chen et al., 2018). Here, drugs are loaded using either the co-crystallization or one-pot method. This method allows for simple drug loading, but each drug molecule must be optimized. Also, this method referred to as one-step encapsulations, this process is accomplished either during MOF synthesis or by utilizing drugs directly as MOF linkers. Additionally, drugs are loaded by two-step encapsulations, which involve impregnation and mechanochemical loading (He et al., 2021).

The encapsulation of drugs within MOFs is motivated by the intricate guest-host interactions established between MOF framework and drug molecules. These interactions encompass a variety of forces, including van der Waals forces, hydrogen bonding, π-π stacking, and electrostatic interactions. The above interactions are pivotal in determining crucial aspects such as loading capacity, release kinetics, and stability of the encapsulated drugs within the MOF. Furthermore, the encapsulation of drugs within MOFs facilitates controlled and sustained drug release, a highly desirable feature in drug delivery applications. The guest-host interactions not only secure the drugs within the MOF but also contribute to the predictability and precision of drug release over time. The high surface area and tunable pore sizes of MOFs open avenues for accommodating a diverse range of drug molecules, encompassing hydrophilic, hydrophobic, and amphiphilic drugs (Mallakpour et al., 2022). Covalent grafting, on the other hand, involves the attachment of chemical functionalities to the surface of MOFs, which can act as entrances for the activated release of the loaded drugs. This method provides a means for controlled drug release by covalently bonding drug molecules or functional groups to the MOF surface, thereby enhancing stability and reducing interactions. Post-synthetic modification in MOFs, particularly cation exchange, has gained significant attention in research due to its potential applications across various domains, leading to the development of novel functional materials. The hard–soft acid–base (HSAB) principle, as described by Hamisu et al., plays a crucial role in guiding experimental clarifications and understanding cation exchange at the secondary building units (SBUs) (Hamisu et al., 2020). This modification allows for the attachment of therapeutic molecules on the MOF surfaces, enhancing stability and reducing interactions.

#### 4.1.1 Co-crystallization

Co-crystallization is the formation of a solid crystal containing both the drug and the MOF. This technique can enhance the drug’s performance, characteristics, including its solubility, stability, and bioavailability (Savjani, 2015). Combining co-crystallization with other strategies, such as the surface coating of MOFs, can improve drug delivery in cancer therapy (Sun et al., 2020). Moreover, this method does not alter the physical and chemical characteristics in the drug, that may be tapped to increase loading efficiency as well as solubility of the drug. For example, drugs that are poorly soluble such as leflunomide (Kritskiy et al., 2019), IBU (Li H. et al., 2017), methotrexate (MTX) (Kritskiy et al., 2020) and lansoprazole (Li X. et al., 2017) have been effectively incorporated into g-CD-MOFs utilizing this method, as well as the drug loading were equivalent if not greater than those of an alternate approach (He et al., 2021). Xui Li et al., investigated that Lansoprazole, a drug molecule, was co-crystallized with γ-CD (gamma-cyclodextrin) and K^+^ in the presence of CTAB (cetyltrimethylammonium bromide) using this method. It involves the formation of a crystalline structure in which the Lansoprazole is incorporated into the cavities of the γ-CD-MOF along with the K^+^ ions and CTAB. This method allows for the creation of a stable complex between the drug and the γ-CD-MOF. The resulting γ-CD-MOF with Lansoprazole had a drug loading efficiency of 21.4 ± 2.3 indicating successful encapsulation of the drug within the MOF structure. This method allowed for the formation of a stable complex between the drug molecule and the γ-CD-MOF, which is desirable for DDS. Overall, the co-crystallization method used in this study demonstrated the successful encapsulation of Lansoprazole within the γ-CD-MOF (Li X. et al., 2017).

#### 4.1.2 One pot method

During the one-pot method, the therapeutic molecule and the MOF are co-precipitated, resulting in uniform distribution of drugs through the MOF’s mesopores (Zheng et al., 2016). When the sizes of pores of the MOF have sufficiently general and inadequate degradation within the MOF regulates release of the MOF, one-pot synthesis was an appropriate technique for preserving the drug molecules (Zhuang et al., 2014; Duan et al., 2017; Munawar et al., 2023). Haoquan et al. synthesized a MOF encapsulating doxorubicin (DOX) using a one-pot process, where ZrCl_4_, terephthalic acid, and DOX were combined in a single step. The resulting MOF exhibited controlled release of DOX and a high drug loading capacity, effectively inhibiting cancer cell growth *in vivo* and *in vitro*. Naser et al. demonstrate that utilizing encapsulated molecules as targets for one-pot synthesis of MOFs for controlled drug delivery in anticancer drug delivery systems (DDS) is a promising approach. Additionally, Zheng et al. present a study showcasing the successful encapsulation of the anticancer drug DOX within ZIF-8 crystals using the one-pot process. The resulting MOF crystals possess hierarchical pores with uniformly distributed mesopores filled with target molecules and ordered micropores inherent to the MOF framework. Importantly, this study highlights the potential of these MOF crystals for controlled drug delivery, particularly in the case of the DOX@ZIF-8 system, which exhibits efficient pH-responsive drug release behavior, making it promising for cancer therapy (Zheng et al., 2016).

#### 4.1.3 Mechano chemical method

It is an eco-friendly method. The mechanical forces produced by the grinding as well as mixing of solid substances in a pestle and mortar may initiate synthesis methods and chemical reactions (Trask et al., 2004; Kaupp, 2009). This drug encapsulation method is easy, quick and effective environmentally friendly (Ding et al., 2022). By grinding drugs such as, p-aminobenzoic acid, 5- FU, caffeine and benzocaine into MOFs, a sustained release and high drug loading amount were obtained (Noorian et al., 2020). Souza et al., developed two separate mechanochemical techniques, namely, automated vortex grinding as well as manual grinding, were employed to achieve encapsulation of 5-FU which is an anti-cancer drug inside the iron-based MIL-100 MOF. The manual grinding approach involved the manual grinding of the reactants using a mortar and pestle, while the automated vortex grinding utilized a standard polypropylene container was coupled to an automatic vortex mixer via a customized holder. These methods allowed for the confinement of 5-FU within the MOF structure through the application of mechanical forces, resulting in 5FU@ MOF composite systems with distinct properties (Souza and Tan, 2020).

#### 4.1.4 Impregnation

The impregnation method includes electrostatic interactions, coordination techniques, and capillary forces to load functional molecules into MOF pores (El-Bindary et al., 2022). The MOFs had been placed in a drug solution that allowed drug molecules to migrate into the MOFs via their porosity. Chemical composition, window dimension, and pore size, liability of MOFs had been crucial factors for drug incorporation success (He et al., 2021). This method is a common encapsulation technique used with CD-MOFs. It involves three steps: i) immersing the synthesized MOFs in solvents or rinsing with solvents and then drying, ii) dissolving guest molecules in suitable solvents or filling them into a confined space, and iii) encapsulating the drug molecules into the activated MOFs. The guest molecules are then absorbed onto the surface of the CD-MOFs through weak interactions such as electrostatic interactions, van der Waals forces, and hydrogen bonding. The resulting CD-MOF/guest molecule composite can then be isolated and characterized (Han Y. et al., 2018). Horcajada et al., synthesized MIL-53 (Fe) using Fe^3+^ as a metal and 1,4-benzene dicarboxylic acid as a ligand was utilized to encapsulate ibuprofen through this method, and the drug loading rate was found to be 20 wt%, suggesting that MIL-53 is an effective carrier for ibuprofen (Horcajada et al., 2008). Jiwen et al., investigated the encapsulation of sucralose into γ-CD-MOF (K^+^) and γ-CD using this method, and the encapsulation efficiency was found to be 27.9% for the nano-sized CD-MOF and 17.5% for the micro-sized CD-MOF. The results suggest that the nano-sized CD-MOF is a more efficient carrier for encapsulating sucralose compared to the micro-sized CD-MOF when using the impregnation method (Lv et al., 2017).

Haiyan et al., investigated that Ibuprofen, a drug molecule, was co-crystallized with γ-CD (gamma-cyclodextrin) and PAA-CD-MOF (polyacrylic acid-modified cyclodextrin metal-organic framework) using both a co-crystallization method and an impregnation method. These methods allowed for the formation of stable complexes between the drug and the MOF materials. The resulting drug loading efficiencies of 12.7 and 13 for PAA-CD-MOF and γ-CD-MOF, respectively, indicate the successful encapsulation of Ibuprofen within the MOF structures. High drug loading efficiencies are desirable for improved drug delivery and therapeutic efficacy. Overall, the study demonstrated the potential of co-crystallization and impregnation methods for drug delivery applications, specifically in the context of Ibuprofen and MOF materials (Li H. et al., 2017).

### 4.2 Direct assembly method

The interaction between a cargo and MOFs are controlled by coordination bonds. As organic linkers, pro-drug or drug molecules may exhibit to the creation of MOFs by coordinating with clusters or metal ions (He et al., 2021). The chemical compounds including essential amino acids, organic linkers, peptides, porphyrins, nucleobases, including drugs, saccharides, and proteins had the capacity to coordinate by metal ions to produce MOFs (Ding et al., 2022). To encapsulate DOX inside the MOFs, Yao et al. (2021) used a direct assembly method, which involved mixing DOX and the MOF precursor in a solvent and then heating the mixture to form MOF crystals. The resultant DOX-loaded MOFs demonstrated a sustained drug release, high drug loading capacity, and synergistic effects of chemotherapy and chemo dynamic therapy on cancer cells.

### 4.3 Post-synthesis method

The molecules of cargo occupy the surfaces of MOFs. This method implies coordination as well as covalent bonds within organic linkers/metal nodes and utilized cargo. It has no effect on the MOF frameworks. A second possible of this technique is adsorption in MOF surfaces. The dominant forces within adsorption typically involve weak interactions that are Van der Waals, hydrogen bonding, and π–π interaction (Lou et al., 2019; Ding et al., 2022). By applying polymers, diverse biomolecules, and ligands, among other molecules, and enhancing the modification conditions, many techniques for modifying the surface of MOFs were investigated (Wang et al., 2018; Katayama et al., 2019; Forgan, 2020). The surface modification of MOFs improves drug loading enrichment and water stability, thus altering the degradation pattern and regulating drug release (Maranescu and Visa, 2022). Paclitaxel (PTX) was loaded into MIL-100 (Fe) through a post-synthesis encapsulation technique in order to reduce PTX’s side effects and increase its efficacy in cancer therapy. PTX has been added to a suspension of MIL-100 (Fe) in ethanol then stirred for several hours to allow the PTX to diffuse into the MOF pores. Resulting PTX-loaded MIL-100 (Fe) demonstrated sustained drug release and increased cytotoxicity against MCF-7 breast cancer cells (Razavi et al., 2022).

### 4.4 *In situ* encapsulation

**FIGURE 8:**
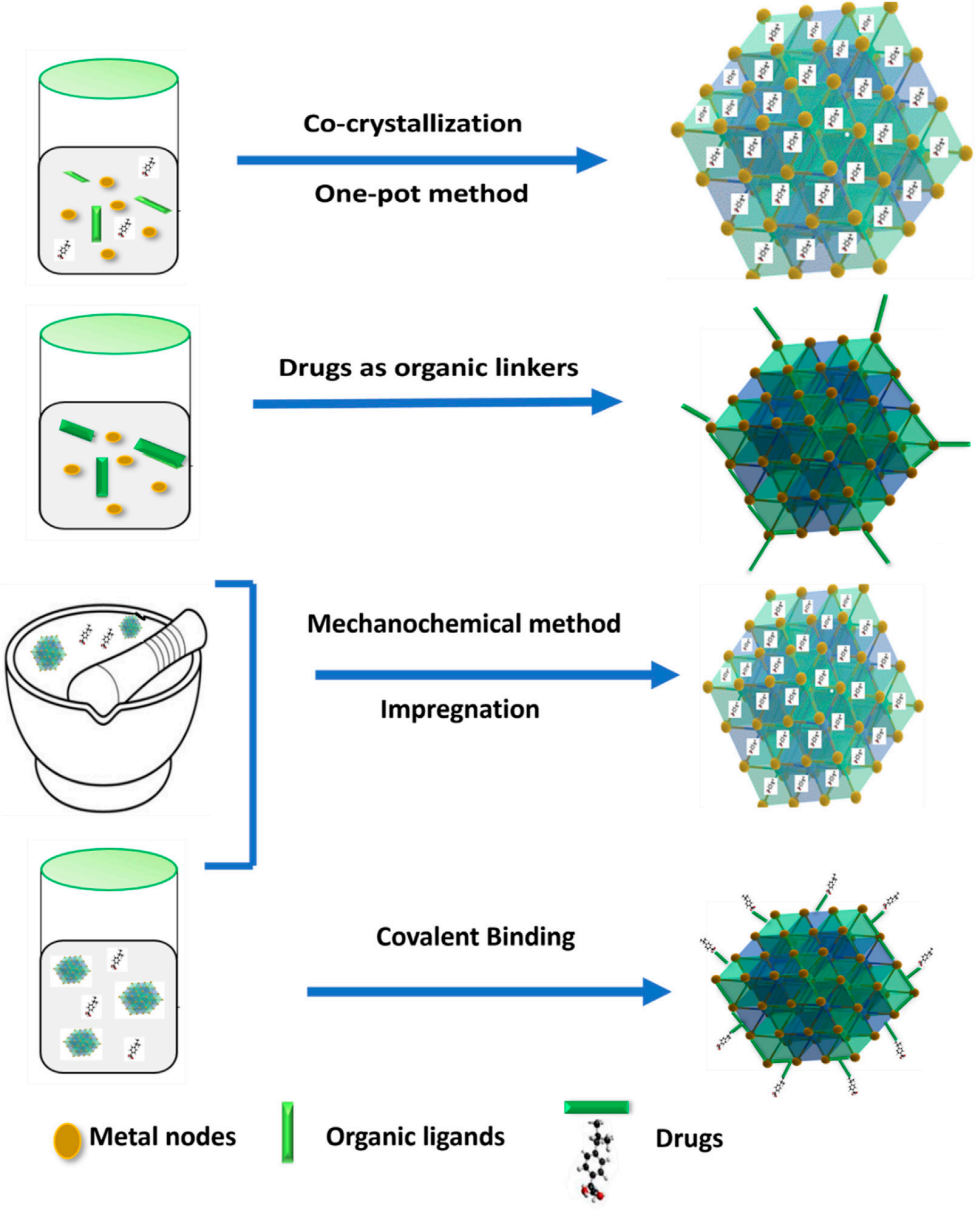
Schematic illustration of the strategies for drug loading into MOF.

In contrast to alternative methods, the *in-situ* formation for a drug-loaded MOF at ambient temperature eliminates both high temperatures required for the synthesis as well as the time required for loading the drug via diffusion. This means that the process is faster and more efficient, making it highly desirable for applications involving drug delivery (Motakef-Kazemi et al., 2014). Anticancer drug doxorubicin (DOX) was encapsulated within MOF composites using an *in situ*, one-step encapsulation technique in aqueous media. The final DOX-loaded MOF composites exhibited controlled DOX release in exposure to external stimuli, for example, pH changes. This method demonstrated a large drug loading capacity contrast to conventional methods as well as could be used in cancer therapy for the controlled delivery of DOX (Adhikari and Chakraborty, 2016). Figure 8 demonstrates the strategies for drug loading into MOF. The selection of the most suitable encapsulation method for incorporating drugs into MOFs for drug delivery depends on various factors such as the nature of the drug, desired release rate, and the specific application requirements. A comparison between the encapsulation methods is presented in Table 2.

**TABLE 2:** Comparison of the encapsulation methods.

Method	Advantage	Limitation	References
Co-crystallization	Simple and efficient method for incorporating drugs into MOFs during the crystallization process	Limited to drugs that can co-crystallize with the MOF components and may not offer precise control over drug loading and release kinetics	Raheem Thayyil et al. (2020), Chezanoglou and Goula (2021)
One-pot method	Offers a straightforward and single-step approach for drug encapsulation within MOFs	Limited control over drug loading and distribution within the MOF structure, and may result in lower drug loading efficiency	Zheng et al. (2016), He et al. (2021)
Mechanochemical method	Provides a solvent-free and environmentally friendly approach for drug encapsulation, and can offer good control over drug loading	Limited to certain types of drugs and MOF structures, and may require optimization for specific drug-MOF combinations	Martí-Rujas (2020), Raptopoulou (2021)
Impregnation	Versatile method suitable for a wide range of drugs and MOFs, and allows for precise control over drug loading, convenient and widely used	May require additional steps for stabilization of the drug within the MOF, and could result in non-uniform drug distribution	Lv et al. (2017), Han et al. (2018b)
Direct assembly method	Offers precise control over the positioning of drugs within the MOF structure, leading to tailored release profiles	May be more complex and time-consuming compared to other methods and could be limited by the compatibility of drugs with the assembly process	Yang et al. (2016b), Lawson et al. (2021), Maranescu and Visa, (2022)

## 5 Functionalization

Functionalization in cancer drug delivery refers to the modification of nanoparticulate DDS with various targeting ligands, imaging agents, diagnostic agents, and other functional groups to improve their specificity and efficacy in drug delivery to cancer cells. This demonstrates the outcomes of synthesizing MOFs with desirable properties (Fatima et al., 2023). MOFs are able to deliver to the lesion sites and flushed out of the bloodstream via passive EPR effect due to their circulation time, ability to evade the immune system, longer and high biocompatibility. A drainage system and poor lymphatic in the cancer cells improve the accumulation of nanoparticles at the tumors (Wang P. et al., 2022). Nanoparticles, ranging in size from 8 to 100 nm, are capable of moving through the tumor via both target-specific and large pores, utilizing a passive targeting mechanism (Fang et al., 2011). Due to the leaky vasculature and poor lymphatic drainage of tumors, MOFs, can accumulate in tumor tissues due to this effect (Yan et al., 2021). These are oftenly designed with the suitable size and surface chemistry that accumulate in tumor tissues, capitalizing on the EPR effect (Yang et al., 2021). MOF accumulation in tumor tissues can enhance the anticancer drug delivery to the malignant site while decreasing their toxicity to healthy tissues (Gu and Meng, 2021). Combining MOFs with other strategies, including functionalization, to enhance their targeting ability as well as drug delivery performance can also enhance the EPR effect (Gu and Meng, 2021). Also by inserting substituent functional groups which include hydroxyl, pyridyl, bromide, methyl, amino, and ethylene into the backbone that act as bridging ligands, MOFs can be functionalized (Cai et al., 2020b). Surface functionalization possesses numerous more additional advantages, such as i) phase transfer, passing nanoparticles through single solvent to different solvent, such as moving from an organic solvent to water; ii) avoidance nanoparticle aggregation; iii) permitting nanomaterials to interact with specific biological molecules of interest, such as nucleic acid, for imaging in delivery; iv) alteration utilizing dyes with fluorescent to obtain specific functionality (Sperling and Parak, 2010).

Targeting modification involves altering the surface of nanomaterials like MOFs to actively target specific sites within the body for drug delivery. Ligands, aptamers, and antibodies are examples of targeting moieties that interact with specific receptors at the site of action, enhancing drug accumulation and therapeutic effects. For instance, glycol polymer-functionalized MOF-808 nanoparticles have been developed for cancer-targeted drug delivery of floxuridine and carboplatin, allowing drugs to be delivered specifically to tumor cells overexpressing specific receptors, thereby enhancing delivery efficiency and specificity (Duman et al., 2022). Biomimetic modification, achieved by coating MOFs with cell membranes, improves their properties. Examples of feasible cell membranes for this modification include cancer cell membranes, platelet membranes, erythrocyte membranes, hybrid membranes, and white blood cell membranes. For example, Zr-based MOFs (PCN-224) loaded with tirapazamine (TPZ) and coated with the membrane of 4T1 cancer cells have been developed for tumor-targeted photodynamic therapy (PDT) and bio-reductive treatment amplified by hypoxia. The membrane coating allows immune evasion, selective tumor accumulation, and homotypic cancer targeting. These MOFs generate cytotoxic reactive oxygen species (ROS) in the presence of visible light and hypoxia, resulting in enhanced anticancer effects (Zeng et al., 2023).

This is essential to alter the surface of MOFs with moieties, such as PEG, CDs, PDA (Ma et al., 2021) to avoid premature release of drugs while enhance their therapeutic effects. MOFs have a high cargo-loading capacity and can reach lesion sites via passive targeting; however, drug accumulation by this method frequently results in poor medical outcomes. By modifying the surface for MOFs with moieties, they can efficiently and actively target their destinations. This enhances the accumulation and therapeutic effects of the drug (Ma et al., 2021). PEG-functionalized UiO-66-NH_2_ MOFs have been designed for the delivery of DOX specifically to tumor cells. The PEG functionalization enhances the biocompatibility and stability of the MOFs and enables selective drug delivery to cancer cells. The UiO-66-NH_2_ MOFs possess a functionalized with a variety of targeting ligands to improve their specificity and potency in drug delivery to cancer cells and high drug loading capacity (Mallakpour et al., 2022). Supplementary Table S1 shows a recent overview of reported MOFs in cancer drug delivery.

The utilization of HKUST-1 (Cu) in biomedical applications has been constrained by concerns surrounding its toxicity and stability, attributed to its hydrolytic instability and the presence of toxic Cu (II) ions. Despite these limitations, HKUST-1 has shown promise in proof-of-principle studies, particularly in magnetophoretic therapy. For instance, Silvestre et al. demonstrated the growth of HKUST-1 layers on magnetic silica nanobeads through liquid phase epitaxy, resulting in a composite proposed for drug delivery (Silvestre et al., 2013). Expanding on this concept, researchers have increasingly incorporated FDA-approved Fe_3_O_4_ nanoparticles into MOFs to address concerns regarding biocompatibility and water stability. Notably, Yang et al. developed a “Litchi-like” Fe_3_O_4_@MIL-100 (Fe) composite, showcasing similar results (Yang et al., 2017; Bellusci et al., 2018). Given the partial FDA approval of Fe_3_O_4_-based drug formulations, the integration of these nanoparticles into MOFs streamlines regulatory pathways and enhances the likelihood of commercial acceptance and progression to human trials (Ke et al., 2019). Moreover, the inclusion of these nanoparticles offers advantages in biocompatibility and stability, essential for successful drug delivery applications. This regulatory approval also signifies rigorous evaluation, positioning MOF composites featuring Fe_3_O_4_ nanoparticles as promising candidates for further development and translation into clinical settings. Additionally, the use of MOF composites containing biocompatible metals like Zn, Fe, or Zr, coupled with organic linkers, underscores considerations for biocompatibility and potential toxicity profiles, enhancing their safety profile for biomedical applications. The use of (Hashemipour and Ahmad Panahi, 2017) MIL-100 (Fe) in drug delivery, particularly for anticancer drugs like DOX, exemplifies the significant potential MOFs in targeted cancer therapy. MIL-100 (Fe) is a MOF composed of iron clusters interconnected by organic linkers, providing a highly porous structure with a large pore size and high surface area conducive to drug encapsulation. Researchers have successfully loaded MIL-100 (Fe) with DOX, taking advantage of its high drug loading capacity to efficiently encapsulate DOX molecules within its pores. The controlled release properties of MIL-100 (Fe) enable the selective delivery of DOX to tumor sites, minimizing off-target effects and enhancing therapeutic efficacy (Silvestre et al., 2013). Furthermore, by functionalizing MIL-100 (Fe) with targeting ligands or stimuli-responsive moieties, researchers can enhance its specificity and control over drug release, making it a promising platform for targeted cancer therapy. The biocompatibility and biodegradability of MIL-100 (Fe) further contribute to its potential as a safe and effective drug delivery system for cancer treatment (Simon et al., 2019; Hu et al., 2022).

## 6 Qualitative and quantitative of MOF for anticancer drug delivery

### 6.1 Qualitative method

Qualitative methods in MOF drug delivery include characterizing and evaluating MOFs based on their chemical and physical properties and their interactions with biological systems (Halamoda-Kenzaoui et al., 2021; Lawson et al., 2021).

#### 6.1.1 Surface properties

**FIGURE 9:**
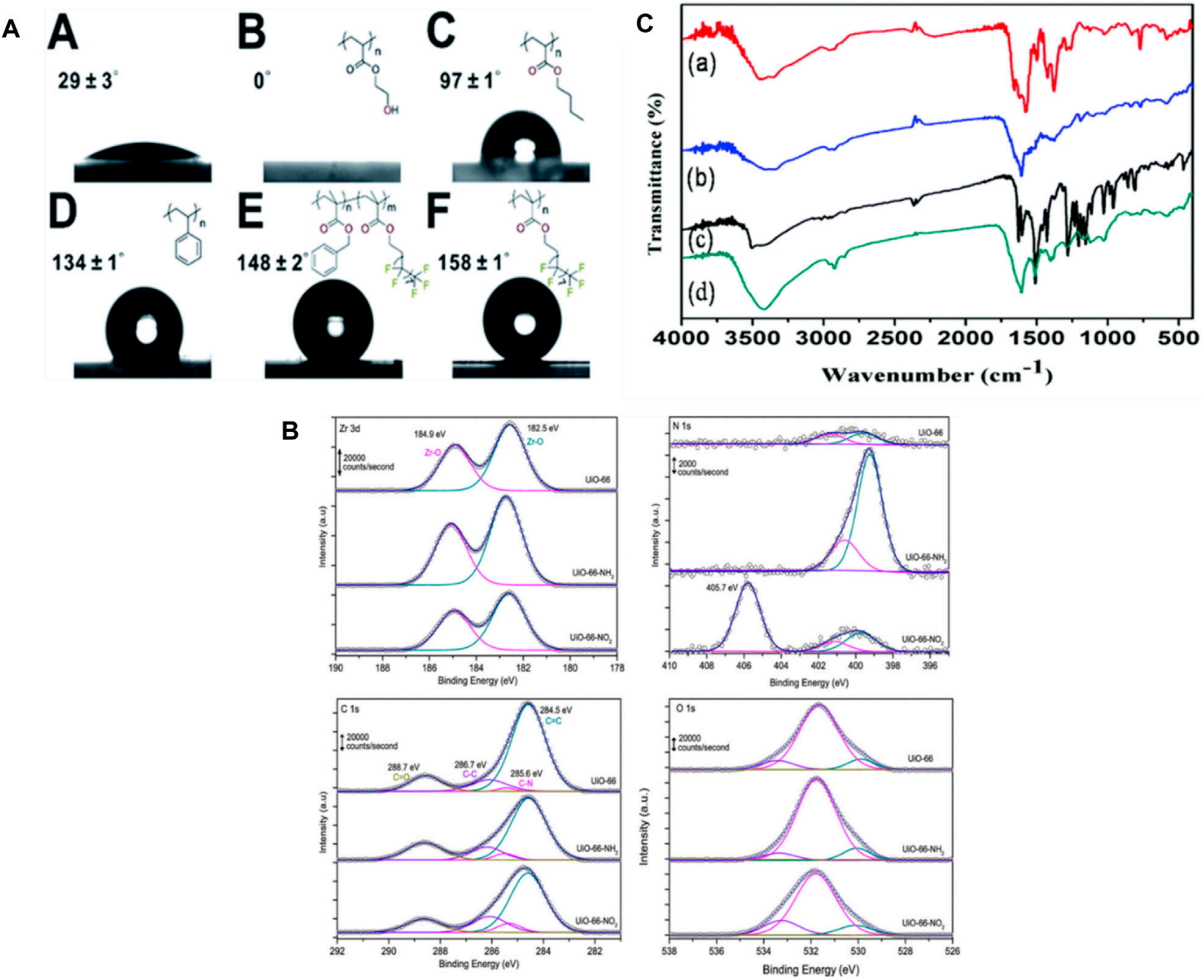
**(A)** Water contact angles were measured for A. UiO-66, B. UiO-66@PHEA, C. UiO-66@xPBA, D. UiO-66@xPS, E. UiO-66@xP (BzMAco-PFMA), and F. UiO-66@xPPFMA (He et al., 2019), **(B)** XP spectra were obtained in the Zr 3d, N 1s, C 1s, and O 1s regions for the three samples evaluated (Farrando-Pérez et al., 2022), **(C)** FTIR spectra were recorded for (a) IRMOF-3, (b) IRMOF-3@FA, (c) IRMOF-3@CCM, and (d) IRMOF-3@FA@CCM (Laha et al., 2019).

Employing techniques such as contact angle measurements, X-Ray Photoelectron Spectroscopy (XPS) and Fourier Transform Infrared Spectroscopy (FTIR), the surface properties of MOFs can be characterized. Such strategies provide data on the chemical composition, functional groups, and wettability of the MOF surface, which may impact their interactions with biological systems (Yang and Yang, 2020; Saeb et al., 2021). To evaluate the surface wettability and its impact on drug loading and release the Wang et al., investigated the water contact angles of various MOFs, such as UiO-66@PHEA and UiO-66. Figure 9A depicts various MOF@ polymer composites in which the wettability of the last composite materials is carefully calibrated by applying different polymer coatings. By integrating a hydrophilic coating, the wettability of the MOF can be decreased, allowing it to become super-hydrophobic. This provides information regarding the hydrophobicity or hydrophilicity of the surface, revealing their interaction with water. This data is essential for designing MOFs with controlled release kinetics and optimal drug loading capacity (He et al., 2019).

Farrando, et al., utilized XPS to examine the surface composition of the synthesized MOFs. Figure 9B indicates that the Zr 3d spectrum exhibits two well-distinct contributions at 184.9 and 182.5 eV, aligning with the Zr 3d_5/2_ as well as 3d_3/2_ contributions, accordingly. The XPS measurements confirmed that the evaluated MOFs contain N, C, Zr, and O. After functionalization regarding N-based polar compounds, nitrogen content significantly increased. Furthermore, the Zr/C ratio was additionally calculated as an indicator of potential structural defects. The functionalized UiO-66 samples contain more Zr, which is most likely attributed to the existence of defects caused by missing linkers (Farrando-Pérez et al., 2022). Using XPS to characterize MOFs allows researchers to gain insight into the surface chemistry, composition, and bonding states of MOFs (Sancho-Albero et al., 2023).

FTIR spectroscopy provides information regarding the functional groups and molecular interactions present on the surface of the MOF. Laha et al. explored this technique to investigate the effects Of Folic Acid (FA) and Ciprofloxacin (CCM) on the surface properties of MOFs. FTIR spectra of the MOFs were obtained, involving IRMOF-3@CCM, IRMOF-3@FA@CCM, IRMOF-3, and IRMOF-3@FA. The analysis revealed characteristic peaks corresponding to the functional groups present in each MOF, indicating successful functionalization of the surface which is indicated in Figure 9C. FTIR measurements can also be used to evaluate the surface modifications of MOFs following drug loading or release. In the case of IRMOF-3@FA@CCM, the highest distinctive peaks of FA and curcumin are evident. Their findings indicate that FA and CCM have effectively attached to IRMOF-3 (Laha et al., 2019). By utilizing FTIR spectroscopy in the characterization of MOFs, researchers can gain valuable information about the surface functional groups, molecular interactions, and changes induced by drug loading or release.

#### 6.1.2 Drug loading and release

**FIGURE 10:**
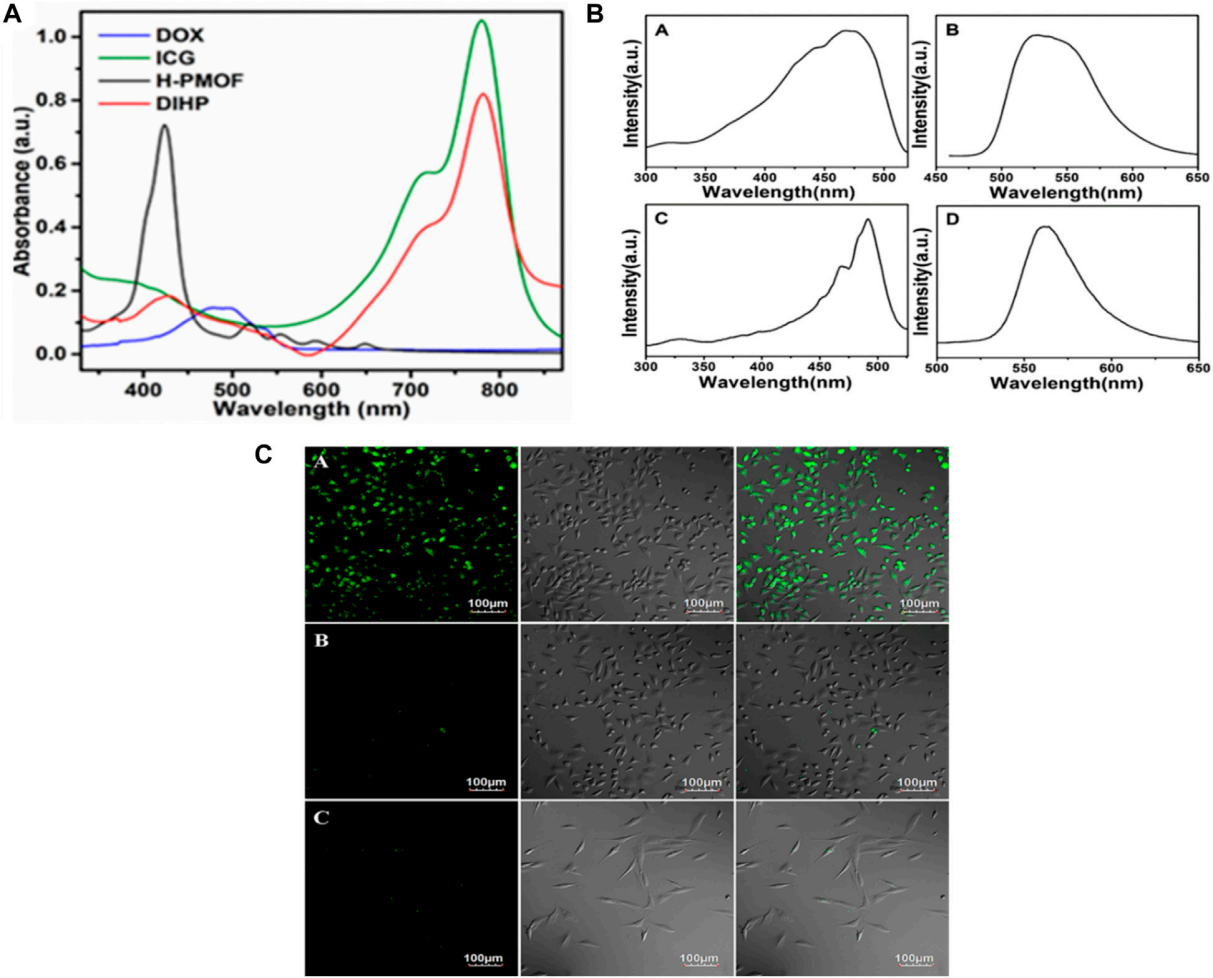
**(A)** Absorbance characteristics in the UV−vis spectrum for free DOX, free ICG, H-PMOF, and DIHP NPs. Excitation (A) and emission, (B) spectra for 5-FAM, and the excitation, (C) and emission, (D) spectra for DDS Fe-MIL-53-NH_2_-FA-5-FAM/5-FU “Reprinted with permission from (Sun et al., 2021).copyright{2021}American Chemical Society,” **(B)** Live MGC-803 cells were subjected to fluorescence imaging during cultivation with Fe-MIL-53-NH_2_-FA-5-FAM/5-FU (A) and Fe-MIL-53-NH2-5-FAM/5-FU, (B) and HASMC cells cultured with Fe-MIL-53-NH_2_-FA-5-FAM/5-FUm (Li L. et al., 2020), **(C)** For a duration of 4 h, the left, middle, and right panels depict dark-field images, bright-field images, and overlays, respectively. The scale bar remains unchanged: “Adapted with permission from (Gao et al., 2017).copyright (2017) American Chemical Society.”

Drug loading and release properties of MOFs can be characterized by Liquid Chromatography (HPLC), UV-Vis spectroscopy, High-Performance and fluorescence spectroscopy. These methodologies offer crucial information for optimizing the DDS regarding the release kinetics, drug loading capacity and stability of the MOF-drug complex (He et al., 2021; Saeb et al., 2021). Using UV-vis spectroscopy, Xin Sun et al., determined the drug loading of DOX or ICG in H-PMOF nanoparticles. Following combining H-PMOF nanoparticles using ICG and DOX solutions, the absorbance of the resulting precipitated dispersion solution was measured. The output shows that direct proportionality between the concentration of the drug in the solution and the absorbance of the solution. By using this, researchers were able to determine the drug loading of DOX or ICG in H-PMOF nanoparticles by measuring the absorbance of the solution. In Figure 10A UV-vis absorption spectroscopy for DIHP revealed absorption bands at 479 as well as 707 nm, which correspond to the distinctive absorption of ICG and DOX, respectively. It additionally appears that H-PMOF possessed one of the largest drug-loading capacities of all self-assembled porphyrin-based nanoplatforms (Sun et al., 2021).

Han et al., developed five MOFs, including UiO-66, UiO-66-COOH, Zr-NDC, UiO-67, and, and UiO-66-NH_2_assessed their 5-FU drug loading capacity using HPLC. Here, Zr-NDC had the highest drug loading capacity for 5-FU as mentioned in Figure 10B. The HPLC measurement provided crucial information for optimizing the design of MOFs for enhanced cancer drug delivery. In this research, the HPLC analysis was performed using an Agilent LC-20AT instrument (Li L. et al., 2020). Figure 10C shows the fluorescence spectrum of Fe-MIL-53-NH_2_-FA-5-FAM/5-FU and 5-FAM in PBS solution. In this diagram, bright green fluorescence is observed once MGC-803 cells have been incubated with this drug and MOFs. However, MGC-803 cells placed with Fe-MIL-53-NH_2_-5-FAM/5-FU or HASMC cells infused with Fe-MIL-53-NH_2_-FA-5-FAM/5-FU do not exhibit any discernible fluorescence contrast. The analysis indicates that solely the FA-conjugated Fe-MIL-53-NH_2_-FA-5-FAM/5-FU can bind to a target molecule. Nanocomposite exhibits an excellent affinity for cancer cells however little contact with healthy cells, proving DDS’s targeted fluorescence imaging capability. Thus Figures 10B, C suggest that the MOF-based DDS Fe-MIL-53-NH_2_-FA-5-FAM/5-FU could be used for a fluorescence imaging agent for cancer cells (Gao et al., 2017).

**FIGURE 11:**
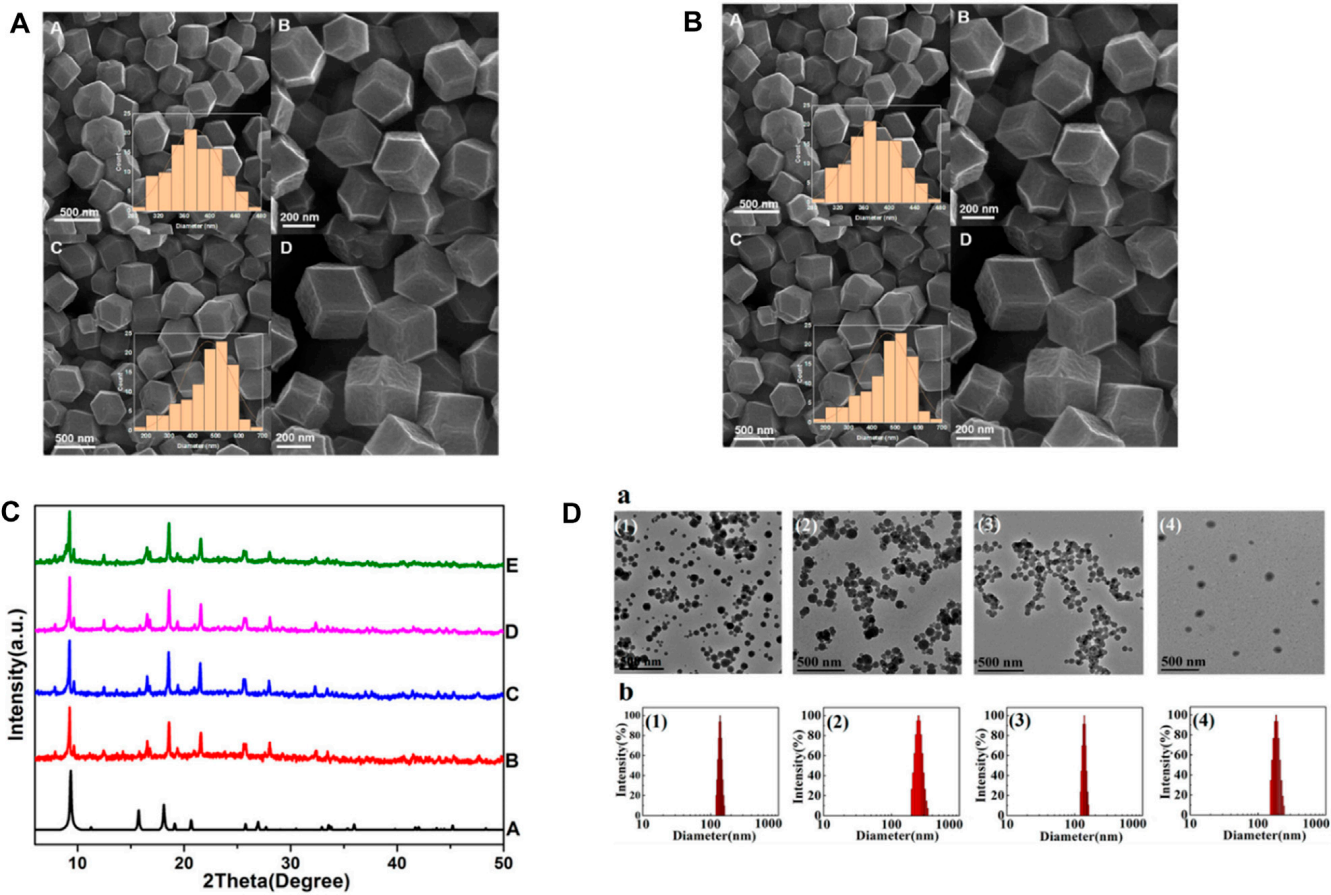
**(A)** FESEM pictures shows the created ZIF-8-GA-CCM using two distinct methods (A and B) at room temperature and (C and D) in an oil bath at 60°C. Insets display size distributions of samples, **(B)** FESEM images of (A and B) ZIF-8-CCM and (C and D) ZIF-8-GA CCM. Insets: size distributions of samples (Khalilian et al., 2023), **(C)** XRD patterns of simulated Fe-MIL-53-NH_2_ (A) and Fe-MIL-53-NH_2_ nanocrystal lines prepared with varying reactant concentrations (B–E) “Adapted with permission from Gao et al. (2017). copyright {2017}American Chemical Society,” **(D)** TEM images (a) size distributions (b) of (1) ZIF-8, (2) DOX@ZIF-8, (3) (DOX + VER) @ZIF-8, and (4) PEG-FA/(DOX + VER) @ ZI`F-8 “Reprinted with permission from (Zhang H. et al., 2017), copyright (2017) American Chemical Society.”

SEM, XRD and TEM can be used to provide information on the MOF’s crystal structure, morphology, and chemical environment, which is essential for comprehending their properties in potential applications (Gu and Meng, 2021; Ding et al., 2022). Figure 11A shows FESEM images of ZIF-8 crystals synthesized under different CCM concentrations. This image shows that the morphology of the crystals changes as the amount of CCM is increased. At low concentrations of CCM, the crystals have a truncated rhombic dodecahedron morphology, while at higher concentrations, the crystals become more spherical in shape. The authors suggest that this change in morphology may be due to the interaction between CCM and the ZIF-8 framework. Figure 11B indicates FESEM images of crystals synthesized with GA and different quantities of CCM. This image shows that the inclusion of GA leads results to increased uniformity and a reduction in the mean particle size of the resulting crystals. Also, authors suggest that this may be due to the role of GA as a cross-linking agent, which helps to stabilize the ZIF-8 framework and promote the formation of smaller, more uniform crystals. Overall, Figures 11A, B provide information on the morphology of ZIF-8 crystals synthesized in the presence of different additives, and demonstrate the potential of FESEM as a tool for studying the structure and properties of MOFs (Khalilian et al., 2023). From Figure 11C Xuechuan Gao et al., explains the XRD patterns of Fe-MIL-53-NH_2_ and simulated Fe-MIL-53-NH_2_ nanocrystalline produced with varying reactant concentrations. The patterns of the synthesized nanocrystalline match well with the simulated pattern, indicating that the synthesized nanomaterials have high crystallinity and the same crystal structure as the simulated Fe-MIL-53-NH_2_. The intensity of the increase’s diffraction peaks with the increase in the concentration of the reactants, which suggests that the crystallinity of the synthesized nanocrystalline structure as the concentration of reactants increases. Overall, the XRD patterns confirm the successful synthesis of nanocrystalline with high crystallinity and the same crystal structure (Gao et al., 2017). From Figure 11D TEM images were used to study the morphology of nanosized MOFs, including ZIF-8, DOX@ZIF-8, PEG-FA/(DOX + VER) @ZIF-8, and (DOX + VER) @ZIF-8. The images demonstrated that all of the samples possessed identical morphology. The size distributions in part (b) show the size distribution of each sample, which was determined from the TEM images. The size distribution is represented by the number of particles versus their size in nanometers (Zhang H. et al., 2017; Khalilian et al., 2023)**.**

MOFs interact with biological systems including cells, nucleic acids and proteins. Isothermal Titration Calorimetry (ITC), Surface Plasmon Resonance (SPR) can be used to characterize these interactions. These methods provide information regarding the binding affinity, specificity, and thermodynamics of the MOF-biological system interaction, which is essential to improve targeted DDS (Halamoda-Kenzaoui et al., 2021; He et al., 2021).

### 6.2 Quantitative method

Quantitative approaches in MOF for anticancer drug delivery encompass the analysis and measurement of MOFs and their interactions with drugs and biological systems. These techniques are essential for improving the DDS, additionally enhancing the drug’s efficacy. The drug loading and release kinetics of MOFs have been evaluated through kinetic studies. In this study, the rate of drug loading and release from MOFs is measured over time and it is essential for optimizing the drug delivery system as well as enhancing the drug’s effectiveness (Cai et al., 2020a; Gu and Meng, 2021).

$$\text{Drug loading efficiency }\left(\%\right)=\left[\left(\text{quantity of overloaded drug}\right)\;/\;\left(\text{total quantity of feeding drug}\right)\right]\times 100\%$$

$$\text{Drug loading capacity }\left(\%\right)=\left[\left(\text{quantity of overloaded drug}\right)/\;\left(\text{quantity of drug overloaded NPs}\right)\right]\times 100\%$$

$$\text{Release Percentage }\left(\%\right)=\left(M_{r}\;/M_{t}\right)\times 100$$

where M_r_ represents released amount of drug and M_t_ indicates the overall amount of loaded drug (El-Bindary et al., 2020; El-Bindary et al., 2022). The maximum drug loading capacity for the produced Fe-BDC-PEG with 5-FU is estimated at 348.22 mg/g complex, achieved at a 5-FU concentration of 10 g/L over 72 h. Approximately 113.44 mg/g of the drug was determined to have been released after 1 hour in the simulated body medium. The drug release rate from the loaded material increased dramatically on day one. Following 7 days in the solution, approximately 92.69% of the drug was released from the material, and after 10 days, 97.52% of 5-FU had been released from the loaded Fe-BDC-PEG complex (Le et al., 2022).

In animal models, pharmacokinetic studies have been used to assess the Absorption, Distribution, Metabolism, Excretion and Toxicity (ADMET) of MOFs. In such investigations the concentration of MOFs in various organs and tissues is measured over time. This statistics is decisive for evaluating the safety and efficacy of MOFs as DDS (Cai et al., 2020a; Saeb et al., 2021). MOF distribution in animal models has been evaluated using biodistribution studies. In such investigations, the concentration of MOFs in various organs and tissues is measured at numerous time points. This data is essential for assessing the efficacy and safety of MOFs as DDS (Saeb et al., 2021). *In vivo* studies, the toxicity of MOFs became scrutinized through toxicity studies. This research measures the effects of MOFs on various organs and tissues, as well as on health and survival in general (Cai et al., 2020a). The effectiveness of MOFs as DDS has been evaluated through *in vitro* experiments which examinations involve exposing cancer cells to MOFs containing drugs and measuring the cells’ viability (Tran et al., 2023).

**FIGURE 12:**
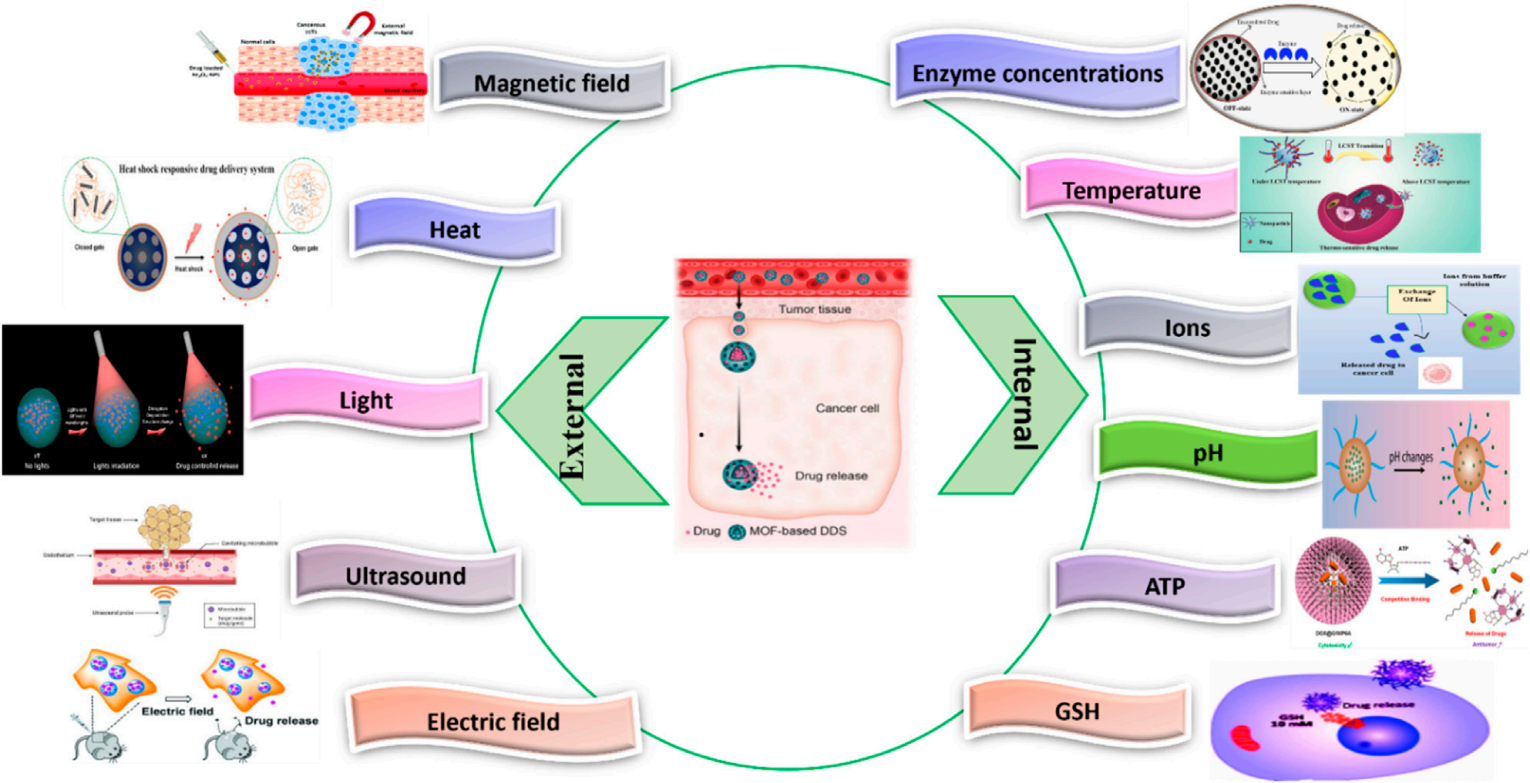
Stimuli responsive MOF (Gao et al., 2010; Ge et al., 2012; Karimi et al., 2016; Cho et al., 2017; Shim et al., 2017; Raza et al., 2019; Yew et al., 2020; Chen J. et al., 2021; Chen M. et al., 2021; Karami et al., 2021; Sanwal et al., 2021).

In DDS, MOF plays a crucial function in the protection and delivery of drugs in the target sites. Some of the examples that have been explored for MOF-based DDS given in Figure 12 including pH, H_2_S, ions, ATP, redox agents, light, heat, enzymes, DNA, enzymes, and disease-specific biomarkers (Wu and Yang, 2017; Zhou Z. et al., 2021; Karami et al., 2021). For instance, a recent study encapsulated 5-FU as a model drug in MOFs, and its H_2_S and pH dual-stimuli responsive controlled release were achieved (Akbar et al., 2022). Another study developed a pH-dependent CS/Zn-MOF@ GO ternary hybrid compound was created, serving as a biocompatible platform for prolonged delivery of 5-FU to human breast tumor cells (Pooresmaeil et al., 2021).

## 7 Applications of MOFs in cancer drug delivery

Owing to their distinct structure, properties and particular features of MOFs are currently devoted to studying these structures in various biomedical applications such as bioimaging, biosensing, disease diagnosis, and drug delivery. MOFs as DDS could improve the release profiles of targeted drugs, increase the drug’s availability at a target site, and permit drugs to be delivered in conjunction with other active agents. It offers unique advantages for targeted and controlled drug delivery, addressing limitations associated with conventional chemotherapy. The usage of MOFs as nanocarriers provides opportunities to develop the efficiency of anticancer drugs, reduce systemic toxicity, and overcome drug resistance. MOFs show some promising properties in anticancer applications, namely, 1) These are utilized as a promising platform for therapeutic nanomedicine, in which the exact vehicle serves as both an imaging (diagnostic) and therapeutic agent. 2) Also, it has been showed to have a tumor-specific killing effect without causing normal cell toxicity. 3) These are applied in the treatment of bone cancer, utilizing radiotherapy to expedite tumor ablation and prevent lung metastasis. 4) It is utilized as dual-drug carriers to improve anticancer effects. 5) For anticancer purposes, MOFs are used as DDS that can react with proteins, peptides, nucleic acids and act as solid supports for bio-entities, thereby improving their stability as well as efficacy (Coluccia et al., 2022). Due to these remarkable characteristics in cancer drug delivery, researchers have been attracted to it recently. In this context, we explain MOFs have applications in cancer drug delivery, including targeted drug delivery, photodynamic therapy, and bioimaging.

### 7.1 Targeted drug delivery

The purpose of targeted drug delivery is transport drugs to particular cells or tissues within the body (Cai et al., 2020a). Drug targeting can be achieved through a variety of mechanisms, including enzyme mediation, use of special vehicles, pH-dependent release and receptor targeting (Ashique et al., 2021). Because of their porous nature, well-defined crystalline structures, and ability to carry high anti-neoplastic agent loadings, it has been investigated as a potential targeted DDS and recently investigated as multifunctional nanocarriers for drug delivery for cancer therapy (Tran et al., 2023). Following steps are involved in the mechanism of targeted drug delivery using MOFs for cancer: Targeting Drug Delivery System (TDDS) is utilized for the specific targeting of drugs to tumor tissues (Cai et al., 2020a). Because of their porous nature, MOFs can transport large amounts of drugs. To increase their specificity towards tumor cells, MOFs can be functionalized with active tumor targeting moieties (Qi et al., 2017). For example, one study reported the development of a mitochondria-targeted MOF which significantly increased the efficiency of a model cancer drug by targeting the drug to the mitochondria of cancer cells (Haddad et al., 2020). MOFs have well-defined crystalline structures which are characterized by a variety of analytical techniques, and their sizes are suitable for regulating drug release *in vivo* (Soomro et al., 2019). It allows, targeted delivery of drugs to cancer cell specifically (Nirosha Yalamandala et al., 2021).

**FIGURE 13:**
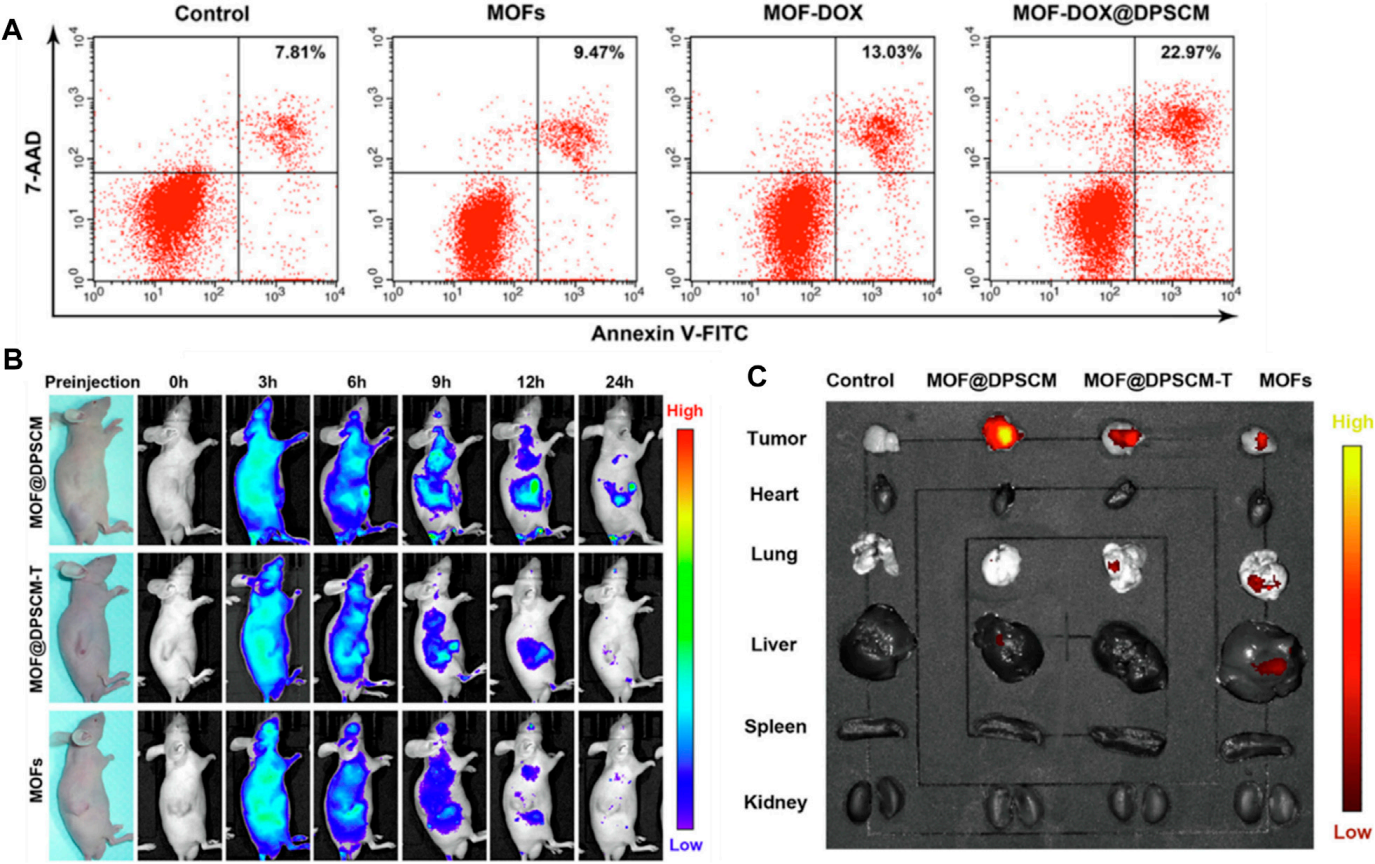
**(A)** Necrosis and apoptosis assays were used to assess the effects of MOFs, MOF-DOX, and MOF-DOX@DPSCM on CAL27 cells *in vitro*. *In vivo* biodistribution of MOFs, MOF@DPSCM, and MOF@DPSCM-T in **(B)** Real-time live fluorescence images of CAL27 mice that had tumors injected via a vein with MOFs, MOF@DPSCM-T, and MOF@DPSCM marked with Cy7 at various time points as well. **(C) (A)** shows *ex vivo* images of the mice’s major tissues. The control group consisted of mice that had been injected with PBS. Reproduced from (Zhou D. et al., 2021) under license from Elsevier.

*In vitro* studies typically involve experiments conducted in a controlled laboratory setting using cell cultures to assess the cytotoxicity, uptake, and intracellular distribution. These studies provide insights into the interactions between MOF and cancer cells, providing observation about the effectiveness and safety of the DDS. In contrast, *in vivo* studies involve experiments conducted on living organisms, such as mice, to evaluate the biodistribution, pharmacokinetics, and therapeutic efficacy. These studies provide a more realistic representation of the DDS performance in a biological system, helping to realize the systemic effects, potential side effects, and overall safety. For example, Zhou et al. conducted *in vitro* experiments to assess the killing activity of MOF-DOX@DPSCM on CAL27 cells. The results showed that MOF alone induced 9.47% apoptotic cells, while MOF-DOX induced ∼13.03% after 4 h. MOF-DOX@DPSCM dramatically induced 22.97% apoptotic cells, significantly higher than any other group. This indicates that MOF-DOX@DPSCM exhibited enhanced killing activity on CAL27 cells which was shown in Figure 13A. Also, they conducted *in vivo* studies to assess biodistribution, therapeutic efficacy, and safety. Cy7-labeled MOF@DPSCM, MOF@DPSCM-T, and MOFs were injected into tumor-bearing mice to assess biodistribution. The results demonstrated that MOF@DPSCM exhibited specific targeting to CAL27 tumor tissue, with higher accumulation compared to MOFs and MOF@DPSCM-T. Figures 13B, C shows biodistribution and *ex vivo* images. *Ex vivo* imaging confirmed the specific retention of MOF@DPSCM in OSCC tissues, indicating its potential for targeted drug delivery. Furthermore, the therapeutic efficacy of MOF-DOX@DPSCM was investigated *in vivo*, demonstrating effective targeting and killing of OSCC cells in the tumor-bearing mouse model, leading to significant inhibition of tumor growth. Pathological evaluation indicated that MOF-DOX@DPSCM eliminated more cancer cells in tumor tissue without causing cytotoxicity in major organs, underscoring its promising value for clinical application (Zhou D. et al., 2021).

**FIGURE 14:**
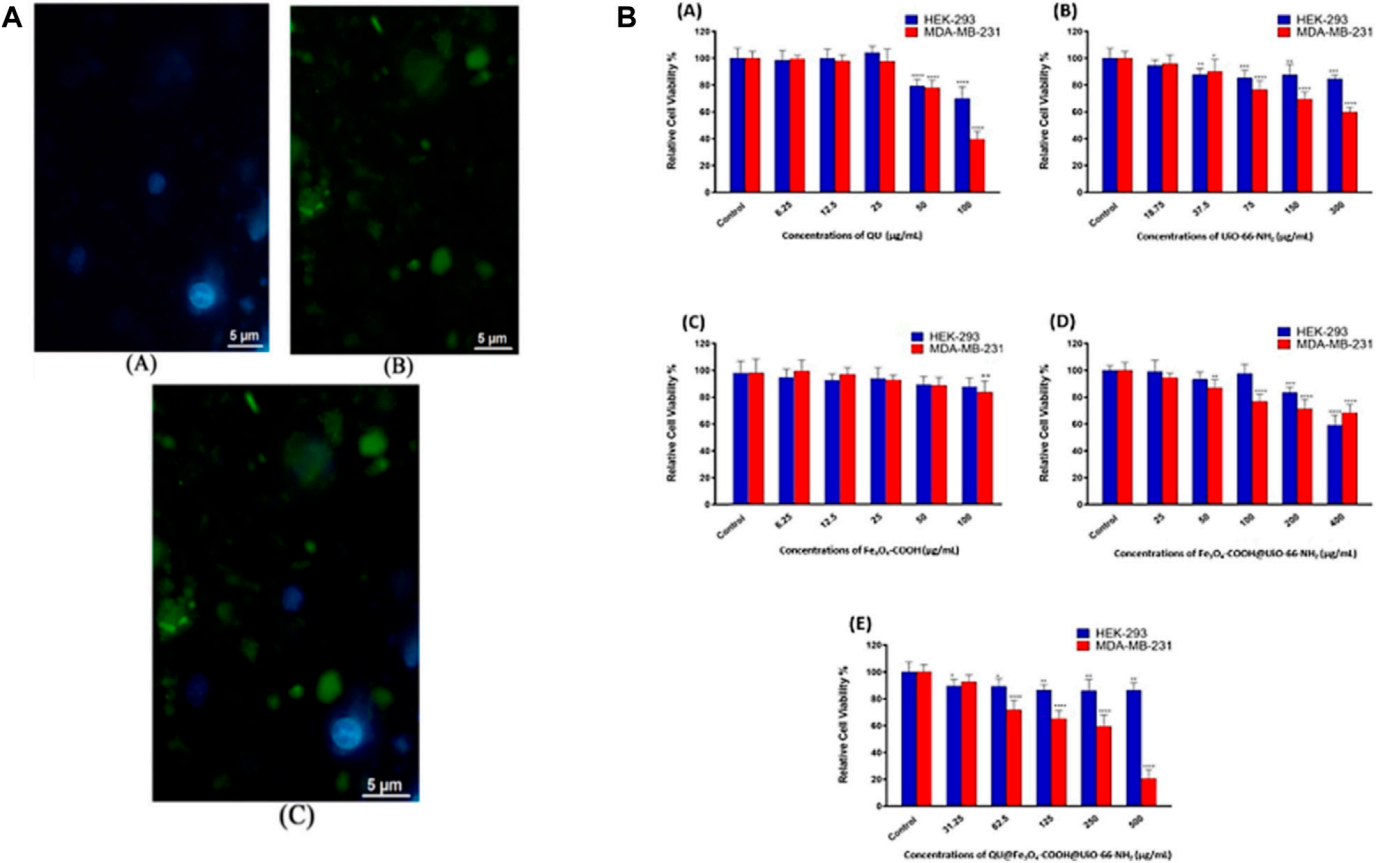
Fluorescence images **(A)** show the A) cell nucleus (blue, DAPI-stained), B) uptake of QU@Fe_3_O_4_-COOH@UiO-66-NH_2_ nanocarrier into MDA-MB-231 cells (green), along with an C) overlay picture (scale bars = 5 μm). **(B)** The cytotoxic effects of (A) QU, (B) UiO-66-NH_2_, (C) Fe_3_O_4_-COOH, (D) Fe_3_O_4_-COOH@UiO-66-NH_2_, and (E) QU@Fe_3_O_4_-COOH@UiO-66-NH_2_ were assessed against both HEK-293 and MDA-MB-231 after a 48-h exposure period (Parsaei and Akhbari, 2023).

Shen, et al., investigated the *invitro* drug release behavior of the nanocarrier at pH 7.4 (normal tissue environment) and pH 5.4 (tumor environment). The nanocarrier exhibited burst drug release initially, followed by prolonged drug release under acidic conditions, indicating on-demand drug release at the tumor site. The cellular uptake of QU@Fe_3_O_4_@UiO-66-NH_2_ was tracked using fluorescence microscopy, showing the internalization of the drug by cancer cells, which was indicated in Figure 14A. The mechanism of cell death induced by the nanocarrier was investigated using Annexin V-FITC/PI staining, showing an increase in apoptotic cells and highlighting the potential of the nanocarrier to induce apoptosis effectively. The cytotoxic effects of QU@Fe_3_O_4_@UiO-66-NH_2_ on human breast cancer cells (MDA-MB-231) were evaluated using MTT assays. The results were shown in Figure 14B. The IC_50_ concentration was determined, and flow cytometry analysis was performed to assess cell viability. Based on the findings, the QU@Fe_3_O_4_@UiO-66-NH_2_ nanomagnetic drug carrier was internalized by cancer cells and triggered cancer cell death via the apoptosis pathway. This nanocarrier exhibited advantageous characteristics including easy and cost-effective production, excellent stability, high drug loading capacity, spacious pore size and extensive surface area, minimal harm to normal cells, and pH-responsive release over a prolonged period. These attributes position it as a highly encouraging DDS with significant promise (Parsaei and Akhbari, 2023). Shen et al., focused on *in vitro* evaluations of the NH_2_-MIL-101(Fe)@GO (MG) composite DDS for colorectal cancer treatment. Various *in vitro* assays were conducted to assess the system’s efficacy and mechanisms of action. The cell cytotoxicity assay revealed that MGD (NH_2_-MIL-101(Fe)@GO@Drugs) exhibited potential to accumulate at the tumor site and interact effectively with tumor cells, leading to reduced cell viability. Additionally, the ROS and apoptosis factor detection studies demonstrated that MGD enhanced ROS production and upregulated the expression of Caspase-3 and Caspase-9 in RKO cells compared to MG alone. The wound healing assay provided insights into the impact of MG and MGD on cell migration, while the ROS releasing assay measured the release of ROS in treated cells. Furthermore, the effects on protein expression of Caspase-3 and Caspase-9 were investigated, revealing alterations in the levels of these apoptosis-related proteins in RKO cells treated with the composite system. Overall, these *in vitro* studies shed light on the potential efficacy and mechanisms of the NH_2_-MIL-101(Fe)@GO composite DDS for colorectal cancer therapy (Shen et al., 2024).

### 7.2 Photodynamic therapy

**FIGURE 15:**
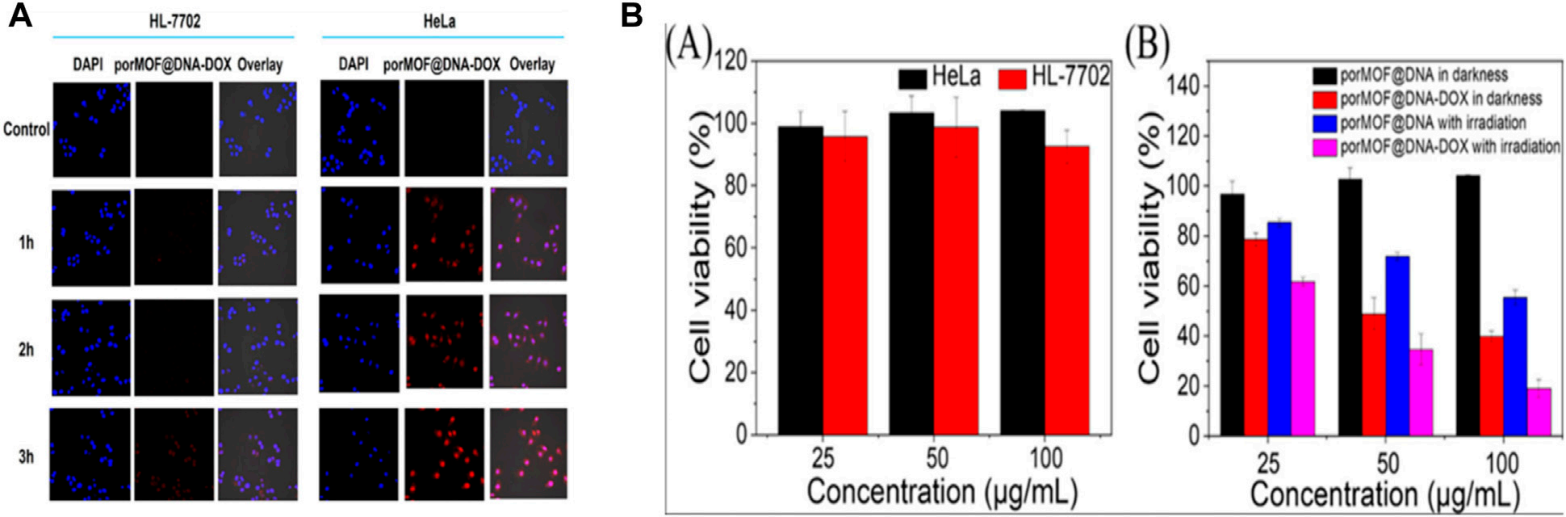
Indicates the **(A)** Fluorescence images were taken of HL-7702 and HeLa cells after incubation with 50 μg/mL porMOF@DNA-DOX for 1, 2, and 4 h **(B)** Cell viability was assessed using MTT assays. (A) Cell viability of HL-7702 and HeLa cells was determined once 18 h of treatment in darkness with varying concentrations of porMOF@DNA. (B) HeLa cell viability was tested following treatment with various therapeutic modalities, which includes porMOF@DNA in gloom, porMOF@DNA at irradiation, porMOF@DNA-DOX in darkness, as well as porMOF@DNA-DOX with irradiation. “Reprinted with permission from Zhao Q. G. et al. (2023), copyright (2019) American Chemical Society.”

Photodynamic Therapy (PDT) is used to treat cancer and infectious diseases by combining light and a photosensitizer (PSs) to create Reactive Oxygen Species (ROS) which result for cellular damage (Shi et al., 2019). It generates ROS through the following mechanism for the treatment of cancer. MOFs are loaded with PSs, which are light-absorbing molecules that produce ROS upon activation. Upon exposure to a specific wavelength of light, the PSs in MOFs are triggered and produce ROS, which can induce oxidative stress and damage cancer cells. ROS can also initiate a cascade of biological events, including apoptosis, autophagy, and immune response, which can augment the anticancer effects of PDT. It can improve the photophysical properties of PSs, such as near-infrared absorption and intersystem crossing, which can boost ROS production and enhance therapeutic efficacy (Yoo and Ha, 2012; Zhou et al., 2016; Alves et al., 2021; Niculescu and Grumezescu, 2021). It can serve as nanocarriers for other therapeutic agents, such as chemotherapy drugs, that can be co-delivered with PSs for synergistic effects (Song et al., 2021). Qiu-Ge Zhao et al., discusses the development and testing of a DNA-functionalized porphyrinic MOF (porMOF) DDS for bimodal PDT and chemotherapy. The *in vivo* experiments using female BALB/c-nu mice showed that the synergistic therapy group had the best therapeutic effect, inhibiting tumor proliferation and achieving tumor ablation, with good biocompatibility and negligible side effects of the porMOF@DNA-DOX nano system. *In vitro* studies involved cell culture experiments using HeLa and HL-7702 cells to evaluate the dark toxicity and phototoxicity of the porMOF@DNA-DOX nano system. Confocal fluorescence microscopy and MTT assays shown in Figure 15B were used to assess the intracellular delivery of DOX, singlet oxygen generation, and cell viability after treatment with different therapeutic modalities. The results demonstrated the selective delivery of DOX into cancer cells, enhanced killing of cancer cells, and the synergistic contributions of PDT and chemotherapy in reducing cell viability. From Figure 15A we can understand the fluorescence images of HL-7702 and HeLa cells incubated with 50 μg/mL porMOF@DNA-DOX for 1, 2, and 4 h show the effective delivery of DOX into HeLa cells via the porMOF@DNA nanodrug loading system, with the fluorescence intensity of DOX in cells increasing over time (Zhao Q. G. et al., 2023). Elnaz Aghazadeh Asl et al., conducted *in vitro* cell cytocompatibility and cytotoxicity assessments using human breast cancer cell lines (MCF-7) and normal cells (MCF 10A) to evaluate cell viability following treatment with various formulations, including both free drugs and drug-loaded microspheres. The effective uptake of chitosan-coated drug-loaded microspheres by cells indicates their potential for *in vivo* drug delivery applications, warranting further assessment of their efficacy and safety in animal models. Additionally, the scientists assessed the biocompatibility of the chitosan-coated microspheres through cell viability tests, comparing the results with ISO guidelines for non-cytotoxicity. They demonstrated that the chitosan-coated microspheres exhibited reduced toxicity compared to the uncoated nanohybrid, suggesting an enhancement in biocompatibility attributed to the chitosan coating (Aghazadeh Asl et al., 2023). Pegah Sadeh et al., investigated the functionalization of β-Cyclodextrin MOF for drug delivery to cancer cells and utilized MCF, AGS, and NIH/3T3 cell lines to evaluate the efficacy of the DDS. Cell viability was assessed using the MTT assay to determine the cytotoxicity and efficiency of various formulations (β-CD-MOF@CCM, glutamine-β-CD-MOF@CCM, CCM-β-CD-MOF, GNPs, and Gelatin-β-CD-MOF@CCM) over a 72-h period (Sadeh et al., 2024).

**FIGURE 16:**
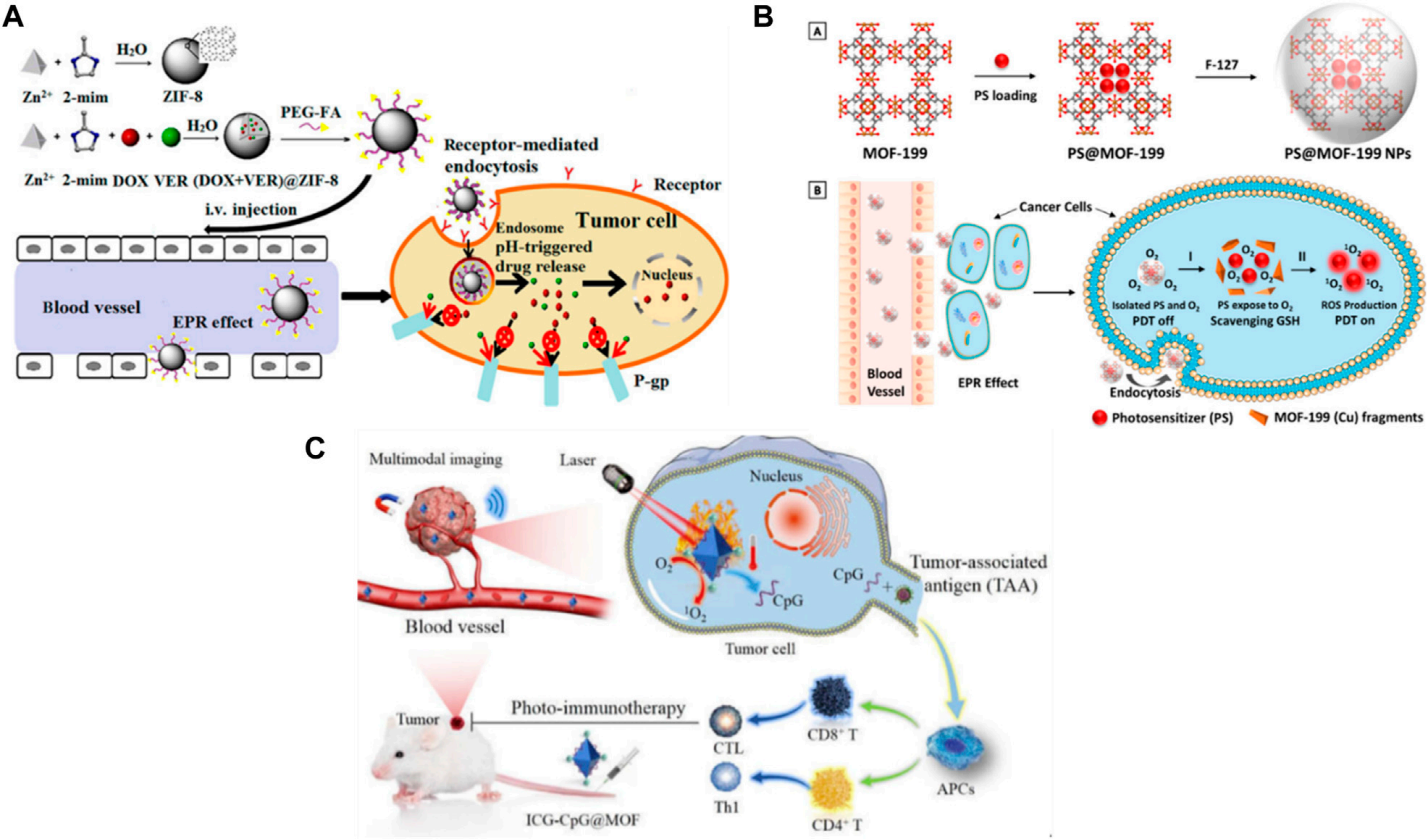
**(A)** Synthesis of PEG-FA/(DOX + VER) @ZIF-8, a pH-Responsive ZIF-8 used as drug delivery vehicles; pH-Sensitive Drug Release in Weak Acidic Environments of Cancer Cells; Accumulation in Cancers via EPR Effect; Integration by Cancer Cells via FR-Mediated Endocytosis; Multidrug Resistant Reversal Mediated by VER “Adapted with permission from Zhang H. et al. (2017), copyright (2019) American Chemical Society,” **(B)** (A) Synthetic scheme to PS@MOF-199 NPs (F127-coated PS@MOF-199) and PS@MOF-199. (B) PS@MOF-199 NPs in the tumor microenvironment were the source of photosensitization inhibition and induction, “Reprinted with permission from (Wang Y. et al., 2019), copyright (2019) American Chemical Society,” **(C)** Represents of A. ICG-CpG@ MOF synthesis and B. Mechanism of multimodality imaging (nuclear magnetic, photoacoustic, fluorescence imaging) guided synergistic cancer photo-immunotherapy. MIL101-NH_2_ is synthesized by heating the mixture of 2-amino-terephthalic acid (NH_2_-BDC) and FeCl_3_ (Fan et al., 2021).

Tang’s et al., created MOF-2 with Cu (II) being the active center as well as porphyrin ligand acting as the PSs, that absorbed GSH and precisely binding with Cu (II), leading to a reduction in intracellular GSH concentration. Subsequently, under light circumstances, it produces an extensive amount of ROS for PDT. Lowering intracellular GSH accelerated cell apoptosis and increased ROS concentrations, improving PDT’s antitumor efficacy. MOF-2 (without light) may exhibit chemotherapy efficacy comparable to camptothecin (CPT), a widely used antitumor drugs. This study revealed MOF-2’s potential as a PDT candidate and tumor prevention agent (Zhang et al., 2018). Zhang, et al., investigated the simultaneous delivery of doxorubicin hydrochloride (DOX) and verapamil hydrochloride (VER) using ZIF-8 nanoparticles decorated with PEG-FA is an example of MOFs serving as nanocarriers for other therapeutic agents in targeted cancer treatment which is indicated in Figure 16A). The ZIF-8 nanoparticles were loaded with DOX and VER, an efflux pump inhibitor that increases intracellular DOX accumulation and overcomes multidrug resistance in cancer cells. The nanoparticles were additionally coated with PEG-FA, which enhanced their stability, biocompatibility, as well as targeting specificity towards cancer cells that overexpress folate receptors. DOX and VER were co-administered using the pH- responsive properties of ZIF-8, allowing the drugs to be released in reaction to the acidic tumor microenvironment. ZIF-8 drug loading ability was high as 40.9%, thereby enhancing the therapeutic efficacy (Zhang H. et al., 2017). Ni et al. recently reported MIL-100 nanoparticles (NPs) loaded with the HA and mitoxantrone. The NPs targeted tumor cells using HA recognition of cluster as differentiation (CD44), and co-injected anti-OX40 antibody changed the immunosuppressive effect, facilitating NPs to enter tumor cells favorably and release chemotherapy drug (Jiang et al., 2021). Siu et al., created a biodegradable mesoporous Fe (III) polycarboxylate MOF with the capability to selectively target the pulmonary region and exhibit pH-sensitive properties for drug administration with the goal to successfully battle the growth of lung cancers (Nirosha Yalamandala et al., 2021). After 24 h, the nanoparticles underwent spontaneous aggregation and subsequent disaggregation in the blood vessels, allowing to the release of the drug. The pH-responsive characteristic can be harnessed for the creation of MOFs which dissolve at a particular pH, that causes rapid drug release and facilitating the delivery of drugs to the correct site within cancer cells (Tran et al., 2023; Zhou et al., 2023).

Liu et al., created PS@MOF-199 by incorporating PSs inside the Cu (II) carboxylate-based MOF-199 which shows in Figure 16B (A). The metal center in MOF-199, specifically Cu (II) might have been utilized as a scavenger for GSH to trigger the activation of the PS and PS activation switch and consume GSH, ensuring that all of the ROS generated by PS were utilized for PDT indicated in Figure 16B (B) (Wang Y. et al., 2019). Zhao et al., utilized Ce6-loaded MOFs, incorporating an organic ligand containing imidazole and disulfide, along with Zn^2+^ metal ions. It is dissolved in acidic organelles facilitating exit via the proton sponge effect resulting by the ionization of the imidazole band. 4T1 cells exposed to Ce6-loaded nanocarriers consumed intracellular GSH via their disulfide bond-thiol exchange reaction regardless of light exposure. Consumption of GSH would result in an increase in cytotoxicity and the inhibition of Glutathione Peroxidase 4 (GPX4). A fully functional nanocarrier has been exhibited *in vivo* antitumor activity in a mouse model with a 4T1 tumor. Because of the combination of iron and chelator ferroptosis inhibitor, the therapeutic impact of the nanocarrier has been diminished. This study demonstrated the impact of ferroptosis triggered by all-active MOF on PDT for antitumor purposes (Meng et al., 2019; Ye et al., 2022). There are still difficulties to overcome, such as toxicity and biocompatibility, drug release prior to reaching the target cancer, and quality control from the laboratory to the industrial scale. Future directions for PDT application in cancer drug delivery using MOFs include improving MOF biocompatibility and specificity, developing more efficient drug release mechanisms, and conducting more *in vivo* studies to evaluate their effectiveness. It has the potential to become a valuable tool in cancer treatment with continued research and development (Lismont et al., 2017; Tran et al., 2023).

### 7.3 Bioimaging

In cancer treatment, MOFs are used as imaging agents and DDS. It exhibit distinct advantages that contribute to their efficacy in cancer treatment and bioimaging (Zhao Y. et al., 2022; Jin et al., 2023). It holds the capability for application in various type of bioimaging techniques, such as single/two-photon fluorescence, up-conversion fluorescence imaging, imaging, and magnetic resonance imaging. It could be together with other nanomaterials to create a particle with multiple imaging features (Zhao D. et al., 2022), also it can be used for a number of biosensing methods, such as Surface-Enhanced Raman Spectroscopy (SERS), electrochemistry, as well as fluorescence. Additionally, cancer biomarkers like proteins, nucleic acids, and metabolites can be found using MOFs (Jin et al., 2023; Munawar et al., 2023). It can be functionalized with fluorescent dyes to allow for fluorescence imaging. MOF distribution and accumulation in cancer cells and tissues can be monitored using fluorescence imaging (Zhao D. et al., 2022). It can be used as MRI contrast materials. Adding targeting ligands to MOFs can increase their specificity for cancerous cells and tissues. Monitoring the buildup of MOFs in cancer cells and tissues using MRI can produce detailed images of soft tissues (Saeb et al., 2021). MRI identifies the electromagnetic wave from an induced gradient magnetic field by measuring varying attenuations caused by the released energy within the material’s different structural atmospheres, which helps to determine the position, nature of tissue and detect alternations in pathology (Slobozhanyuk et al., 2016). These can be used as PAI (Photoacoustic Imaging) contrast materials. Also, it has the ability to absorb light and produce acoustic waves that an ultrasound detector can pick up. In addition to monitoring the buildup of MOFs in cancer cells and tissues, PAI can provide high-resolution images of soft tissues (Jin et al., 2023). It can be utilized as CT contrast materials. Adding targeting ligands to MOFs can increase their specificity for cancerous cells and tissues. When used to monitor the buildup of MOFs in cancer cells and tissues, CT can produce detailed images of internal structures (Zhang M. et al., 2020). pH-responsive ZIF-8 and glutathione have been frequently utilised as bases for the synthesis of microscopic Fe_3_O_4_ nanoparticles to be T1 contrast molecules. This led to the development of Fe_3_O_4_-ZIF-8 to be a T2 contrasting agent (Zhao et al., 2016).Compared to the inherent drawback of a solitary imaging technique, recent advances in amalgamating diverse imaging process may complement one another to provide comprehensive diagnostic data (Wu and Yang, 2017). For instance, Fan, et al., recently developed the ICG-CpG@ MOF multimodal imaging nanoplatform aiming to treat and detect cancer. This nanoplatform uses Fluorescent Signal Donors (ICG) and photoacoustic to cover a particular MOF, MIL101(Fe), which serves to be the platform’s central carrier. The carboxyl-activated ICG was attached to the amino group of MIL101-NH_2_ via an amido bond. Due to porous and electrostatic adsorption, CpG was bonded to MIL101-NH_2_. ICG-CpG@ MOF was enhanced in cancer cells via the EPR effect, and 808 nm laser light was used to activate photoimmunotherapy which is shown in Figure 16C (Fan et al., 2021). The UIO-66-NH_2_-FA-5-FAM/5-FU, designed for fluorescence imaging and a multifunctional system for cancer therapy, was created by Gao et al. According to a study, a microemulsion method that uses nanoscale zirconium-porphyrin MOFs (NPMOF) can produce a biocompatible IGTS. Fluorescence imaging and PDT are possible due to the high porphyrin content (59.8%). Massive amounts of doxorubicin (DOX) were loaded into NPMOFs as a chemotherapeutic drug (109%) and released as a result of pH changes. PDT double systems and fluorescence targeting in the chemotherapeutic is confirmed by the occurrence of NPMOFs at the tumor site following laser irradiation and release of DOX (Gao et al., 2018; Munawar et al., 2023).

## 8 Computational analysis of metal organic framework in drug delivery

Recently, extensive analysis has been performed to comprehend the role of MOF in drug delivery application via experimental techniques, while theoretical aspects were less explored. Computational studies offer atomic-level insights analysis of structure and properties of MOFs, which are difficult to obtain experimentally (Zhang S. et al., 2020; Chen, 2022; Demir et al., 2023). These insights help in understanding the fundamental mechanisms of MOF behavior and optimizing their design for specific drug delivery applications (Erucar and Keskin, 2017). Additionally, a large number of drug-MOF combinations can be screened using molecular simulation to identify probable interactions, these simulations can help reduce the number of potential MOFs for experimental studies, saving time and resources (Mashhadzadeh et al., 2020).

Experimental research cannot be replaced by molecular simulations; rather, they complement each other. Experiments gather macroscopic data from the real world, while molecular simulations mimic the outcomes of experiments by determining the microscopic properties of an atomistic level. The first stage of quantum mechanical (QM) models consists of minor fragments within a periodic MOF as well as it is employed to calculate the interaction between the drug molecules and host MOF. Classical method consists of an entire MOF structure in periodic manner with thousands of atoms as well as, it is utilized to examine the diffusion of drugs and adsorption for MOF structures in the second stage (Tylianakis et al., 2011). To examine the role of MOFs in drug delivery, Density functional theory (DFT), molecular docking, molecular dynamics (MD) simulations, and monte carlo simulations have mostly utilized (Kotzabasaki and Froudakis, 2018). The table below describes the various simulation techniques utilized for theoretical MOF studies. The simulation method used in MOF is indicated in Table 3.

**TABLE 3:** Simulation methodology used in MOF.

Simulation method	Variants	Applications	References
Quantum chemical methods	DFT, Semi-empirical techniques	Docking, potential energy, force field parameterization, and geometry optimization	Merz (2014)
Classical methods	Molecular mechanics (MM), molecular dynamics (MD), simple molecular mechanics force field (FF), Grand Canonical Monte Carlo (GCMC)	Solubility, glass transition, hydrogen bonding, drug aggregation, crystallization, miscibility, and interaction between carrier-drug	Katiyar and Jha (2018)

### 8.1 Molecular docking

Molecular docking in MOFs is a technique that determines the optimal fit between two molecules after a guest molecule (drug) is docked with accessible binding sites within a MOF. Utilizing this techniques, researchers can predict the binding affinity of drugs to MOFs and evaluate the related binding mechanisms, thereby facilitating the screening of materials (Keshavarz and Barbiellini, 2023). While identifying the binding location in MOFs through molecular docking, the framework serves as a macromolecule and the guest as the ligand (Karthikeyan et al., 2023a; Keshavarz and Barbiellini, 2023). This technique can shed a spotlight on the mechanism of drug distribution within MOFs.

**FIGURE 17:**
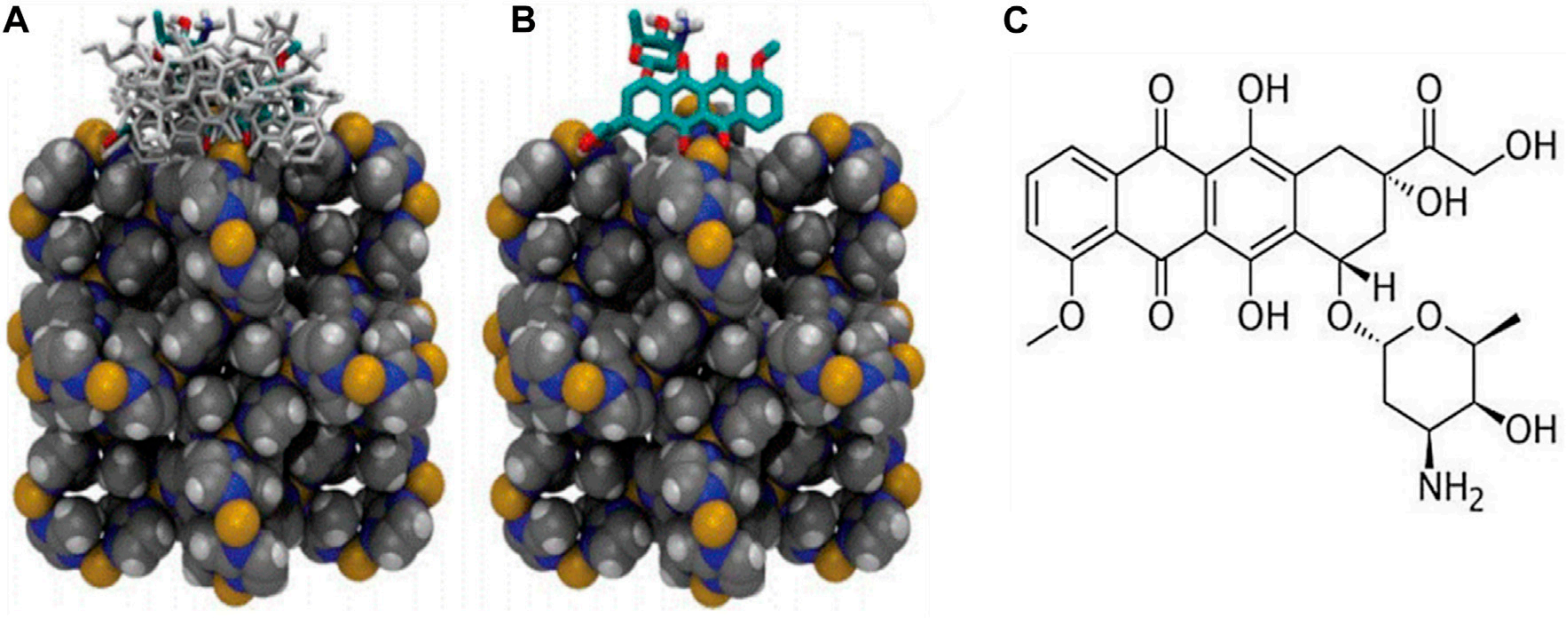
Illustration of the ZIF-8 crystallographic unit cell (represented by van der Waals spheres) and the docked conformations of doxorubicin in stick form. **(A)** Ten lowest energy conformations of doxorubicin bound to the X-ray structure of ZIF-8. **(B)** The lowest energy conformation with an occurrence of ca. 70% among the 100 lowest energy conformers selected from a total of 1.35 × 10^8^ sampled conformations. **(C)** Chemical structure of doxorubicin. Nitrogen in blue, carbon atoms in grey, Zn^2+^ cations in yellow, and hydrogen in white (Vasconcelos et al., 2012).

Lane et al., used this technique to calculate the binding mode and ligand’s affinity (drug molecule-DOX) to a receptor (protein or porous material -ZIF-8). They performed docking to investigate the binding of Doxorubicin (DOX) to ZIF-8. It involved creating a set of DOX-ZIF-8 complex conformations using a search algorithm, and then evaluating and ranking each form using an empirical energy function. The atomic positions of ZIF-8 structure have been derived using crystallographic structures identified under both high as well as normal pressures to determine the material’s potential flexibility. These calculations were conducted using a hybrid search technique depending upon the Lamarckian Genetic Algorithm (LGA) enforced by the AutoDock4 software. To produce accurate measurements of host (MOF)-guest(drug) interaction energies, grid maps with 126 × 126 × 126 dimensions were computed. In addition, to predict the binding affinity and effectiveness of the host-guest interactions, the results of the docking simulations revealed the preferred binding mode of DOX to ZIF-8. The experimental data were interpreted using these findings, and new knowledge about the mechanism of drug release from ZIF-8 was obtained. Ten configurations of DOX attached to the X-ray structure of ZIF-8 with the least energy are depicted in the Figure 17, along with a conformation that occurs among the 100 conformations with the lowest energy (Vasconcelos et al., 2012). The results of docking simulations were used to interpret experimental data and gain insight into the mechanism of drug release from ZIF-8 (Vasconcelos et al., 2012).

**FIGURE 18:**
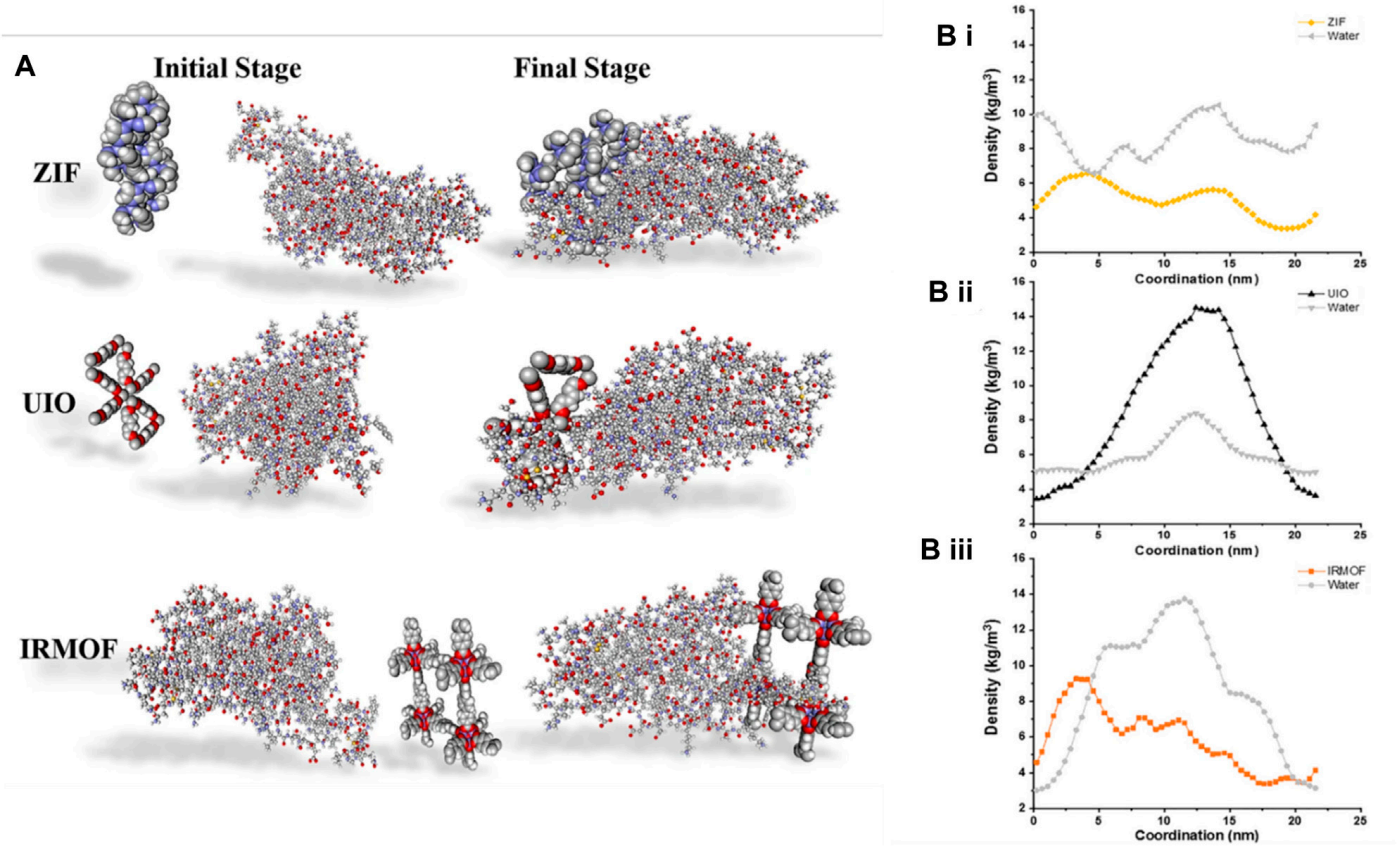
**(A)** The initial and ending snapshots of the interactions between MOF structures and S protein. **(B-i–iii)** Density distribution of MOF structures and water together with simulation boxes for ZIF, UIO, and IRMOFs, respectively (Dahri et al., 2022b).

Dahri et al., were applied LINCS constraints to coarse-grained simulations with 30 fs steps at 3,000 ns, taking into account a cut-off radius of 2 nm and hydrogen bonds (H-bonds). These systems’ simulation boxes measured 20 × 20 × 20 nm. The researchers utilized the Auto Dock Tool-1.5.6 for docking simulations and added the gasteiger charge and polar hydrogen into the ACE2 PDB files and spike (S) protein of SARS-CoV-2, respectively. The files had been subsequently saved in the form of. pdbqt. ACE2 and the S protein docking procedure were then carried applying the autodock-vina-1-1-2-linux-x86 programmed. Figure 18A represents the start and end of the interactions among structures of MOF and S protein. In this context, the water molecules are not considered. Figure 18B reflects density of water and MOF within the simulation boxes. The increased uniform density visual of ZIF across the box validates the S protein’s even distribution of MOF. The distinct peaks of IRMOF and UIO along the planes confirm the vertical arrangement of creates with an inferior S protein interface (Dahri et al., 2022a).

### 8.2 Molecular dynamics

**FIGURE 19:**
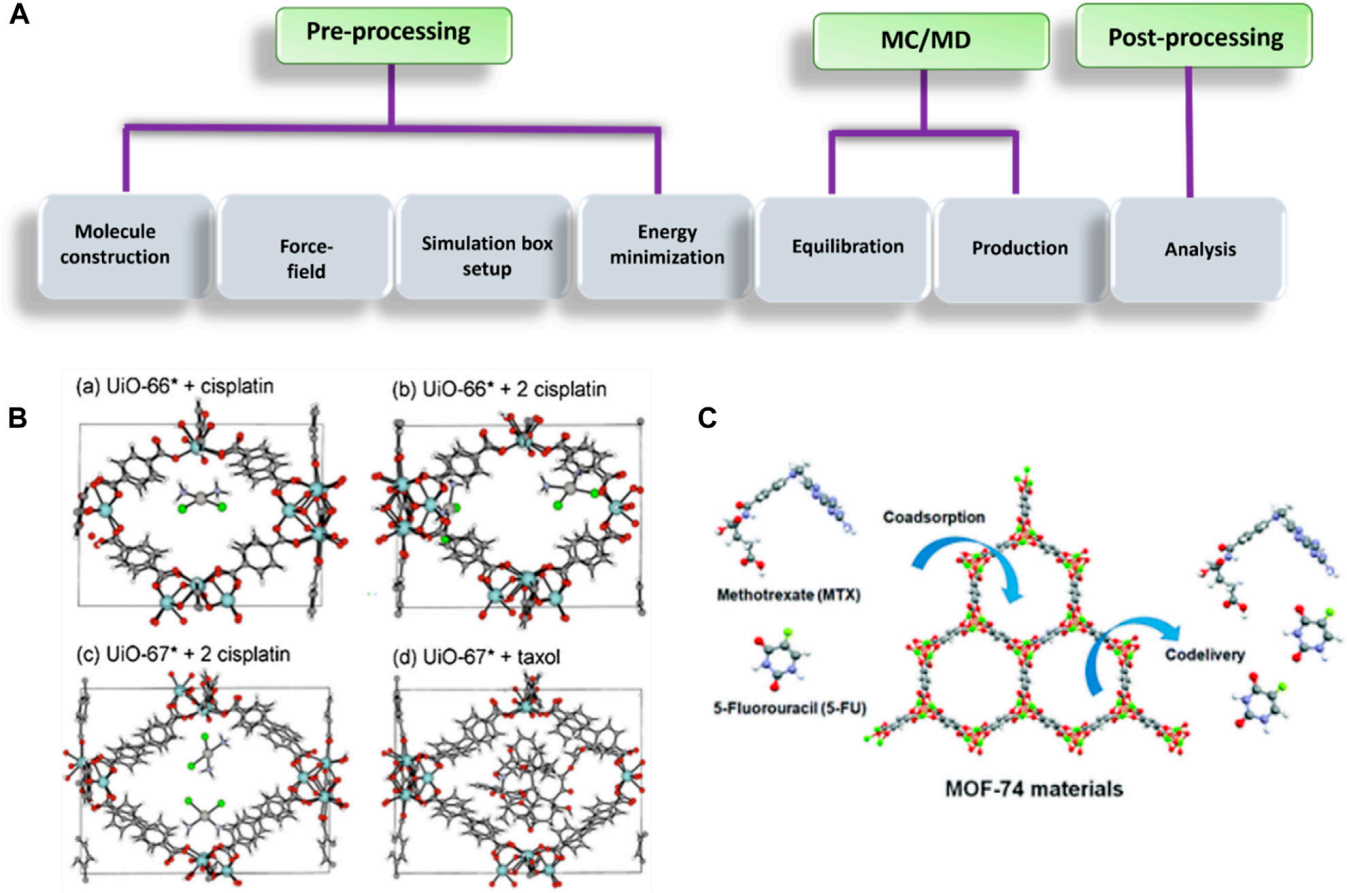
**(A)** Basic scheme for simulation in the field of drug delivery (Katiyar and Jha, 2018). **(B)** After 5,000 fs, MD-simulation visualization Reprinted from Biocompatible Zr-based nanoscale MOFs coated with modified poly(ε-caprolactone) as anticancer drug carriers, 509/1–2, M. Filippousi, S. Turner, K. Leus, P. I. Siafaka, E. D. Tseligka, M. Vandichel, S. G. Nanaki, I. S. Vizirianakis, D. N. Bikiaris, P. Van Der Voort, and G. Van Tendeloo, Int. J. Pharm., 208–218, Copyright (2016), with permission from Elsevier (Filippousi et al., 2016), **(C)** MD simulation of MOF-74 for anti-cancer drug storage and delivery (Erucar and Keskin, 2017).

Simulations of molecular dynamics (MD) are one of the primary computational techniques utilized in drug delivery research (Rupavarshini et al., 2023). It can be used to examine drug diffusion within the pores of MOFs, providing a screening tool for experimental investigation (Karthikeyan et al., 2023a, 2023b). It simulates the motion of atoms and molecules over time using classical mechanics and demonstrates a comprehensive understanding of the release mechanisms and storage in MOFs, allowing to determine the molecular interactions and optimal sites for drug and MOF. Figure 19A illustrates the fundamental scheme of simulations of MD in drug delivery. Liu et al., reported, molecular dynamic studies in three bio-MOFs and one MOF to investigate the adsorption and diffusion of drugs (Liu et al., 2014). Utilizing MD simulation, it can be feasible to take into account of intramolecular and intermolecular interactions to determine the velocity, positions, and forces of the drug molecules (estimated on Newton’s second law) after reaching equilibrium. Simultaneously, the determination of the diffusion coefficients MOF-drug complex (Smit, 2008; De Vivo et al., 2016). Also, it can be used to predict the release rate of drugs from MOFs under diverse thermodynamic conditions in the context of drug delivery. Researchers are able to determine the most favorable sites for drug adsorption and diffusion by comparing the results of MD simulations to experimental data (Bunker and Róg, 2020).

Fillipousi et al., investigated Zr-based MOFs, such as UiO-67*, and UiO-66* coated with altered caprolactone (poly), as promising anticancer carriers for cisplatin delivery. Both MOF structures exhibited drug adsorption, with experimental loadings of approximately 10 mg g^−1^ in UiO-67 as well as 48 mg g^−1^ in UiO-66. Furthermore, simulations showed that taxol filled the void generated by UiO-67* with limited mobility during the entirety MD-simulation, verifying that inorganic brick defects had been necessary for adsorption and diffusion inside the pores of UiO-67*. This study demonstrated the porous, crystallinity and biocompatibility of the polymeric coatings of these Zr-based nMOFs, making them an effective carriers to the controlled delivery of anti-cancer agents (Filippousi et al., 2016). Figure 19B demonstrates the MD-generated snapshots of cisplatin molecule adsorption in UiO-67 and UiO-66 for t = 5,000 fs.

Eura et al., examined the anti-cancer drug delivery capability of MOFs and discovered that MOF-74 can be used as a carrier for two anti-cancer drugs, methotrexate (MTX) and 5-fluorouracil (5-FU) as depicted in Figure 19C. The study found that there were greater interactions between MOF-74 and MTX at lower fugacities, but 5-FU exhibited greater adsorption due to its better entropic effects at greater fugacities. The grid maps used in the study had 126 × 126 × 126 dimensions. The search results also indicate that MOFs have good loading capacity for drugs and can be utilised as carriers in DDS. MOFs offer a unique opportunity for reinventing the landscape for life-saving medications (Erucar and Keskin, 2017). Shahabi et al., examined the effectiveness of peptide-based MOFs (MPF) for delivering the drug 6-mercaptopurine (6-MP) under the influence of an external electric field in a separate MD-based article. They demonstrated the drug molecules possessed strong interactions in MPF at shorter electric field intensities than at greater intensities; therefore, implementing the Electric Field (EF) did not enhance the effectiveness of drug storage. Promoting an ability of the EF led to an increase in dynamic motions as well as decrease in the coefficient of diffusion, highlighting the negative impact of EF on interaction between molecules (Shahabi and Raissi, 2019; Mashhadzadeh et al., 2020).

### 8.3 DFT

DFT is one of the computational techniques for examining the electronic properties and characteristics of materials, including MOFs. It involves approximations and numerical methods to solve the Kohn–Sham equations and calculate the electron density, which is then used to determine the various properties of MOF. It can be utilized to determine both MOF-drug complex interactions and its binding energy. This data can be applied to the design of MOFs with high drug loading capacities as well as the optimized release rates (Kotzabasaki and Froudakis, 2018). This calculations can precisely describe host–guest interactions, adsorption sites between guest drug molecules and host MOFs.Also, it identifies the best favorable conformations which applied to the study of anti-cancer drug delivery (Horcajada et al., 2008; Babarao and Jiang, 2009). The optimal conformation of each cluster was computed as an initial point for the subsequent DFT geometry optimization. By performing these calculations, researchers can gain insights into the electronic properties, bonding interactions, and other characteristics of MOFs, which can be valuable in designing and optimizing these materials for specific applications (Ernst et al., 2023).

**FIGURE 20:**
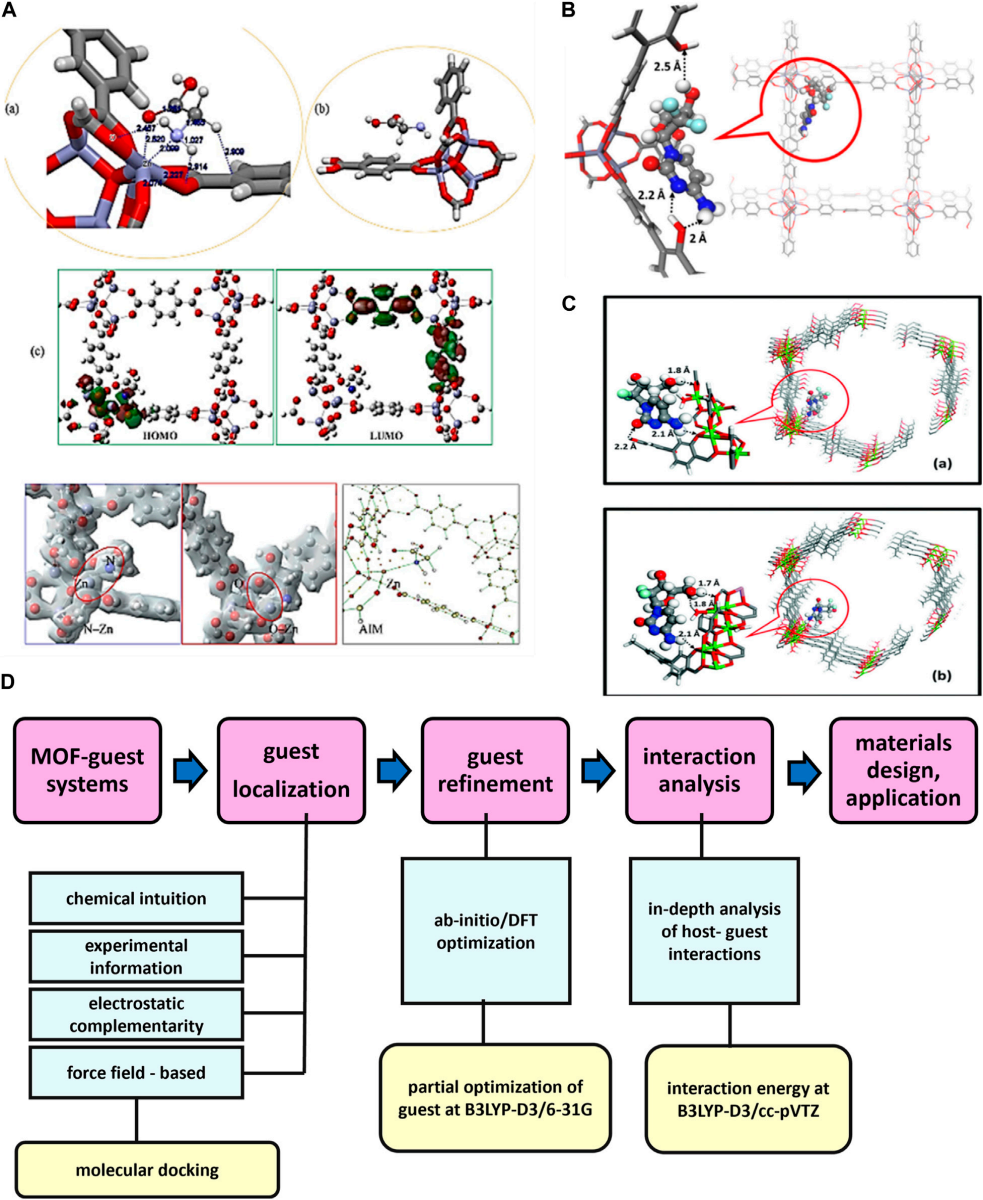
**(A)** Illustration of (a) the optimized geometry of energetically preferable configuration of glycine/MOF-5 and (b) selected portion of the optimized complex for MP2/aug-cc-pVTZ calculation. Calculated (c) HOMO/LUMO orbitals (d) total charge density and (e) bonding critical point (BCP) for energetically stable state of glycine/MOF-5. Reproduced from (Mostafavi et al., 2022) under license from Elsevier, **(B)** The most stable configuration of OH-IRMOF-16 molecular fragment with GEM as determined from DFT calculations. Reproduced from Kotzabasaki et al, (2017b) under license from Elsevier, **(C)** Optimized geometries of GEM with respect to (a) the OH-IRMOF-74-III and (b) IRMOF-74-III molecular fragments as those derived from DFT calculations (Kotzabasaki et al., 2017a). **(D)** The computational location and analysis of guests in MOFs.

Using this calculation Mina et al., reported the interaction between the amino acid glycine and MOF-5. This research employed various techniques to enhance hydrogen bonding interactions between them to discover the optimal adsorption sites for glycine. Figure 20A depicting the electronic structure and the adsorption energies of glycine in MOF-5 were used to illustrate the results of the DFT calculations. Through these calculations, the bond lengths within the atoms in the MOF-5/GLY complex have also been found. The optimizedMOF-5/GLY complex has been determined to be the supreme stable structure depending upon the calculated interaction energy of −45.251 kcal/mol (−1.962 eV). The optimal distance between the nearest atom of the MOF-5 and glycine molecule (the Zn atom of the framework and the N atom of glycine) proved approximately 2.099 Å. Here, in an aqueous medium, the observed interactions and the electronic structure nature are also analyzed. The interacting systems were evaluated for long-distance dispersion corrections. This studies demonstrated that the feasibility of calculations in the design of MOFs with enhanced drug loading and release properties (Mostafavi et al., 2022).

Figure 20B depicts the remarkably enduring arrangement of the OH-IRMOF-16 molecular fragment based on geometry of gemcitabine (GEM). Here the high binding energy indicates that the strong interaction between drug and framework, suggesting that OH-functionalized IRMOF-16 has the potential to serve as a drug carrier. For instance, the binding energy is −22.6 kcal/mol, demonstrating a strong interaction between drug and OH-IRMOF-16 structure. Figure 20B illustrates the optimized structures of GEM attracting onto two distinct MOFs: a) OH-IRMOF-74-III and b) UiO-66-NH_2_. Through DFT calculations, the active sites were identified between MOF and drug, also the binding energy and loading capacity of the frameworks were determined. In OH-IRMOF-74-III, hydrogen bonds among the oxygen atom of MOF and the amine, hydroxyl groups of the GEM preserve the strong interaction between the carbonyl group of the GEM and the hydroxyl unit of the OH-linker. Thus, applying calculations, researchers have investigated the adsorption mechanisms of drugs onto MOFs. DFT can provide knowledge about how drugs interact to the MOF surface by analyzing the electronic properties and reactivities of drug molecules (Kotzabasaki et al., 2017b).

Marianna et al., reported DFT calculations to identify adsorption sites and the most favorable conformations for a drug called GEM in a porous nanocarrier called IRMOF-74-III. Figure 20C shows that it provides a visual representation of the most stable drug configurations when compared to the fragments of molecules of OH-IRMOF-74-III and IRMOF-74-III. Figure 20C shows how the MOF structure creates an extra interaction site with GEM upon addition of an aromatic hydroxyl group to it. Additionally, estimated binding energy among OH-IRMOF-74-III and GEM is higher than that among IRMOF-74-III and GEM. This suggests that the incorporation of the aromatic hydroxyl moiety to organic linker of IRMOF-74-III creates an extra interaction site with GEM, which could potentially enhance drug adsorption and delivery (Kotzabasaki et al., 2017a).

Michelle Ernst et al., reported graphical representation of the computational identification and analysis of guests in MOFs. Figure 20D highlights their methodology in yellow (Ernst et al., 2023). The computational techniques which have been applied drug delivery field to study the interactions between drugs and MOFs, have potential applications in DDS. In particular, DFT calculations can provide fundamental information about a material’s properties, while molecular docking calculations can predict the binding affinity and orientation of a ligand to a receptor, and molecular dynamics simulations could provide information into the stability and dynamics of the ligand-receptor complex over time. Overall, these simulations might offer insights into the behavior of drugs and materials in DDS, which can aid in the design and optimization of these systems. Thus, these computational methods contribute to the understanding, design, and optimization of MOFs for drug delivery applications, whether individually or in combination.

## 9 Conclusion

In this comprehensive review, we delved into the fascinating world of MOFs and their groundbreaking potential in cancer drug delivery. Initially discovered in the late 1990s, MOFs have emerged as promising candidates for drug delivery due to their tunable pore structures, high surface areas, and versatile chemistry. We highlighted the advantages of MOFs over traditional organic and inorganic nanomaterials, emphasizing their ability to precisely control drug release kinetics, enhance therapeutic efficacy, and minimize off-target effects. Through the exploration of synthesis, functionalization, ADMET, EPR effect, encapsulation, factors affecting performance, stimuli-responsive behavior, bioimaging, photodynamic therapy, targeted drug delivery, autodocking, autodynamics, and DFT simulations, we have unveiled a promising avenue for revolutionizing cancer treatment. MOFs offer a unique opportunity to co-deliver multiple drugs within a single framework. This opens up new avenues for combination therapies, where synergistic drug combinations can be precisely administered, targeting different aspects of cancer biology simultaneously for enhanced efficacy.

The innovative design and tunable characteristics of MOFs enable efficient encapsulation and controlled release of therapeutic agents, offering a new frontier in personalized medicine. Their high surface area, unique pore structures, as well as versatile chemistry provide unparalleled opportunities for drug loading, enhancing therapeutic efficacy while minimizing adverse effects. By harnessing the EPR effect, MOFs can selectively target tumor tissues, ensuring precision drug delivery and sparing healthy cells from unnecessary toxicity. Various synthesis methods for MOFs were explored, including steam-assisted convention, sono chemical, mechanochemical, hydrothermal, solvothermal, and electrochemical methods, each offering unique advantages in terms of scalability, reproducibility, and control over particle size and morphology. We delved into the modification of MOFs for cargo loading, including encapsulation, co-crystallization, one-pot methods, mechanochemical methods, impregnation, direct assembly methods, post-synthesis methods, and *in-situ* encapsulation, with illustrative examples provided to elucidate each approach. Furthermore, the integration of stimuli-responsive elements allows for on-demand drug release triggered by specific cues within the tumor microenvironment, optimizing treatment outcomes.

The application of MOFs in bioimaging offers a non-invasive means of real-time tracking, facilitating accurate diagnosis and treatment monitoring. Additionally, their compatibility with photodynamic therapy contributes to enhanced imaging capabilities through multimodal approaches. Functionalization strategies for MOFs were discussed, focusing on enhancing their specificity and efficacy in anticancer drug delivery. Qualitative and quantitative assessment methods for evaluating MOFs in drug delivery applications were outlined. *In vitro* studies involve assessing cellular uptake, cytotoxicity, and intracellular drug release using cancer cell lines. These studies provide valuable insights into the biological behavior of functionalized MOFs and their potential for targeted drug delivery. *In vivo* studies are crucial for validating the therapeutic potential of functionalized MOFs in animal models of cancer. These studies involve evaluating pharmacokinetics, biodistribution, tumor accumulation, and therapeutic efficacy following systemic administration of functionalized MOFs loaded with anticancer drugs. By correlating *in vitro* and *in vivo* data, researchers can assess the translational potential of functionalized MOFs for clinical use in cancer therapy.

Simulations provide a powerful tool for gaining insights into the complex interactions between MOFs and drugs at the molecular level. Through simulations, we gain valuable insights into the intermolecular interactions within the MOF-drug complex, laying the foundation for further optimization and rational drug design. Furthermore, Density Functional Theory (DFT) is a computational method commonly used to study the electronic and structural properties of materials, including MOFs. By applying DFT simulations, researchers can calculate the electronic structure, energy levels, and bonding properties of MOFs, providing valuable insights into their performance and reactivity. This information is crucial for rational drug design and optimization of MOF structures for specific drug delivery applications. Using simulation techniques, these properties are calculated as an average over sufficiently large ensemble collections or extensive simulation times to comparison with experimental results. Finally, by collecting identical data using atomistic scale models, it is achievable to discover previously inaccessible thermodynamic details underlying experimental results. To ensure the accuracy of a theoretical model, simulation results should be verified with experimental data. In the end, simulations can frequently be transferred between similar systems to rapidly generate vast amounts of data, which is especially helpful when screening the MOFs for drug delivery.

The review highlights the potential of MOFs in cancer drug delivery, which is a rapidly developing field that combines cutting-edge science and medicine. MOFs are versatile and adaptable delivery platforms that can be functionalized to target cancer cells specifically. They have been shown to enhance drug loading and delivery, improve pharmacokinetics and biodistribution, and reduce side effects. The review discusses the recent advances in MOF-based drug delivery systems, including the use of bioMOFs, which are particularly suitable for controlled delivery in biological applications. The review also highlights the challenges and limitations of MOF-based DDS, such as the need for further studies on their biodistribution, degradation kinetics, and accumulation in tissues and organs. Overall, the review underscores the potential of MOFs in cancer drug delivery and their promise in advancing precision medicine and personalized treatments.

## 10 Future perspectives

Developing MOFs with numerous functions, such as targeted drug delivery, imaging, and combination therapy, could result in more effective anticancer treatment strategies. It would be essential for the clinical translation of MOFs to address their safety, toxicity, and manufacturing aspects. The safety and efficiency of MOFs for anticancer drug delivery in clinical settings could be validated by additional animal models and human clinical trials. The majority of studies have primarily concentrated on the drugs biodistribution and bioavailability following oral/intravenous treatment, with a notable gap in understanding the comprehensive pharmacokinetics of MOFs as part of the ADMET process. Regarding bioavailability, there is a critical need to shift attention towards enzymatic degradation (such as CYP450) and efflux transporters (like P-gp) which inhibit drug absorption and prevent leakage from the body. Since MOFs possess a number of benefits, there have remain a number of tasks that must be undertaken before they are able to be used for clinical use, which identifying the solubility and toxicity of MOFs in the body and conducting extensive research on the biocompatibility of MOFs. To decrease the toxicity of MOF-based DDS, researchers must focus primarily on biocompatible MOF selection, surface modifications and functionalization, controlled drug release, biodegradable MOFs, computational modeling, preclinical evaluation, combination therapy, clearance pathways and long-term safety. Our future studies encompass a comprehensive approach from both clinical and preclinical perspectives. Firstly, we aim to design and synthesize a biocompatible MOF by carefully selecting safe metal ions and ligands, ensuring minimal cytotoxicity and immunogenicity for cancer drug delivery. Additionally, we plan to explore the development of core-shell MOF structures, leveraging the core for drug encapsulation and the shell for surface functionalization with targeting ligands to enhance specificity towards cancer cells. Furthermore, our research will focus on optimizing drug loading strategies within the MOF, aiming for efficient encapsulation and controlled release of therapeutic agents. In the future, we intend to conduct *in vitro* studies to assess the cytotoxicity, cellular uptake, and therapeutic efficacy of MOF-based DDS using cancer cell lines. Subsequently, we will proceed to *in vivo* studies using animal models to evaluate the biodistribution, pharmacokinetics, and therapeutic outcomes of the developed MOF formulations. In parallel, computational studies utilizing techniques such as molecular docking, molecular dynamics simulations, and DFT calculations will be employed to elucidate the molecular interactions between MOFs and drug molecules, providing valuable insights into their drug delivery mechanisms and optimization. Additionally, we will investigate ADMET properties of MOF-based DDS to assess their safety profiles and potential for clinical translation. Overall, our future studies aim to advance the understanding and application of MOFs in cancer diagnosis and therapy through a multidisciplinary approach combining experimental and computational methodologies.
