# Supplementary material for: MOFs for next-generation cancer therapeutics through a biophysical approach—a review
Source: Front Bioeng Biotechnol. 2024 Jun 13;12:1397804. doi: 10.3389/fbioe.2024.1397804 (PMC11208718; doi:10.3389/fbioe.2024.1397804)
Supplement: Supplementary file 1 [file Table1.docx]

**Supporting Information**

| **Metal** | **Organic ligand** | **Drug** | **MOF name** | **Stimuli/**  **Functionalization** | **Cell Type** | **Applications** | **Outcome** | **Reference** |
| --- | --- | --- | --- | --- | --- | --- | --- | --- |
| Zn | 2-methyl imidazolate (2-MIMs) | Zinc phthalocyanine  (ZnPc) | ZnPc@ZIF-8 | pH | HepG2 | Photodynamic therapy (PDT) | (ZnPc) exhibited high photodynamic anticancer efficacy in vitro, highlighting the potential of ZIF-8 as an excellent DDS for delivering photosensitizers (PS) in PDT and providing new opportunities for the development of effective cancer therapies. | (Song et al.,2018) |
| Zr | TCPP | Doxorubicin  (Dox) | PCN-224 | Enzyme (HAdase) | CD44, MDA-MB-231, SCC-7 and HEK 293T | Chemo and  Photodynamic therapy | Greater therapeutic efficacy when exposed to light. Consequently, the result shows system with the potential for enhanced therapeutic efficacy. | (Kim et al.,2019) |
| Zr | Tetra (4-carboxyphenyl)  porphyrin (TCPP) | - | PCN-224 | - | MCF-7 | Chemo dynamic  therapy | The nanoparticles were discovered to be of an optimal size, biocompatibility, and safety. For combined starvation therapy and CDT, they were able to increase H_2_O_2_ levels and decrease GSH levels, which inhibited tumor growth, prolonged mouse survival, and induced tumor cell apoptosis. | (Zhu et al.,2023) |
| Zr | TCPP | DOX and Indocyanine green (ICG) | (DOX and ICG) H-PMOF | pH | Murine mammary  Carcinoma (4T1) | Chemotherapy  and Photothermal therapy (PTT) | Nanoparticles exhibited exceptional homologous tumor targeting and immune evasion abilities. In addition, the fact that three mice in the study had completely eradicated tumors without complications | (Sun et al.,2021) |
| Ytterbium chloride | 1,1’-Ferrocenedicarboxylic acid | DOX | Fc-MOFs-  Mann | pH | HepG2 | Chemo dynamic therapy | The MOF was able to selectively target HepG2 cells, release drugs in response to changes in pH, and achieve enhanced therapeutic efficacy for cells while minimizing damage to normal cells. This study demonstrates the potential of utilizing MOFs to provide a versatile therapeutic system to the cancer treatment. | (Dong et al.,2022) |
| Zn | 2-MIMs | DOX+VER | (DOX + VER) @ZIF-8 | pH | B16F10 and MCF-7 | targeted cancer therapy. | The uptake of drugs into tumor cells, the accumulation of the enhancement of cell uptake into MDR cells, doxorubicin (DOX) in MCF-7 cells, and inhibition of tumor growth suggest that the study aimed to examine the efficacy of DDS and their potential in overcoming multidrug resistance in cancer cells. | (Zhang et al., 2017c) |
| Zn | 2-methyl imidazole | DOX | ZIF-7, ZIF-8 | upon  contact with biomimetic systems and pH | - | Cancer drug delivery | The experiment examined the potential of ZIF nanospheres as an intelligent drug delivery system for the anticancer drug Doxorubicin. Under various external stimuli, including pH contact and changes with biomimetic systems, controlled release of the drug and the encapsulation using ZIF-7 and ZIF-8 were examined. The results were promising, with ZIF-7 delivering the drug for 10 hours and ZIF-8 allowing for 3 hours of controlled release. | (Adhikari et al., 2015) |
| Fe | 2-amino terephthalic acid | 5-FU | Fe-MIL-53-NH_2_-FA-5-FAM/5-FU | FA (Folic acid) | MGC-803 | Targeted Drug Delivery and Magnetic/ Optical Resonance Imaging | It revealed that the MOF-based DDS exhibited superior biocompatibility and high cellular uptake efficiency in MGC-803 cells. In vitro and in vivo, the DDS exhibited sustained drug release and enhanced therapeutic efficacy. | (Gao et al.,2017) |
| Fe, Al or Zr | 4,4’-dithiobisbenzoic acid (4,4’-DTBA) | Curcumin  (CCM) | CCM@MOF-Zr (DTBA) | pH, High Glutathione (GSH) | HeLa and MDA-MB-231 | Cancer Therapy | Noteworthy antitumor activity of CCM@MOF-Zr (DTBA) in as compared to CCM (chemotherapeutic drug) alone. In rats injected with CCM@MOF-Zr (DTBA), the tumor volume remained nearly unchanged after 14 days, indicating effective tumor growth inhibition. The rate of tumor inhibition for CCM@MOF-Zr (DTBA) was approximately 76.1%, which is significantly higher than the rate of inhibition for free CCM (35.1%). In addition, the body weights of the mice in all three groups were comparable, indicating that the drug carriers were not toxic. | (Lei et al.,2018) |
| Zr | Tetrakis [4-(4-carboxyphenyl) phenyl] ethene (TCBPE) | Doxorubicin hydrochloride | MOF-DOX | - | MCF-7 | Cancer treatment | MOF nanotubes are excellent drug carriers due to their hollow structures and ultrahigh loading capacity. The fluorescence of MOF nanotubes permits the monitoring of drug release in real time. As a result, the production of MOF nanotubes with potential applications in drug delivery and other fields requiring a high loading capacity and real-time monitoring. | (Chen et al.,2021b) |
| Fe | 1,4-benzene- dicarboxylic acid (BDC) | 5-fu | Fe-BDC-PEG@5-FU | Ultrasound | MCF7, AGS and HT-29 | Targeted drug delivery (cancer therapy) | The Fe-BDC-PEG@5-FU system inhibited strongly on breast, gastric, and colon cancer cell lines. With increasing exposure time, the drug-loaded material's inhibitory capacity increased. The breast cancer cell line exhibited the most potent inhibitory capacity. At 120 hours of exposure, the IC50 of Fe-BDC-PEG@5-FU was approximately 3.75 g/mL, which is comparable to the inhibition level of pure 5-FU. After 10 days, the 5-fluorouracil release from the Fe-BDC-PEG material reached a maximum of 97.52 %, according to the study. This suggests that Fe-BDC-PEG@5-FU system has the potential to be an effective cancer drug delivery system. | (Le et al.,2022) |
| Mn | oleic acid (OA) | FOE | DUCNP@Mn−MOF | pH | 4T1 | cancer therapy | The DUCNP@Mn−MOF/FOE system demonstrated significant efficacy in inhibiting tumor growth in vivo, achieving a high tumor growth inhibition rate. By combining chemotherapy with catalytic therapy, the system exhibited synergistic effects that led to a substantial reduction in tumor growth. Moreover, the system displayed excellent tumor-targeting ability and bioavailability, positioning it as a promising candidate for effective cancer treatment. The nanoparticles enabled rapid release of the drug at the tumor site, particularly in acidic tumor microenvironments. The drug release rate reached over 70% within the first hour in an acidic environment, indicating efficient drug utilization and delivery to the cancer cells | (Zhao et al.,2023b) |
| Fe | 2-amino-terephthalic acid | luteolin and matrine | NH2-MIL-101(Fe)@GO | pH | RKO | Cancer treatment | PH-responsive Fe-MOF@GO composite drug delivery system for treating colorectal cancer was positive. The developed MOF carrier efficiently loaded luteolin and matrine, with luteolin exhibiting an acidic pH-responsive release characteristic. The composite system showed enhanced anti-cancer activity by inhibiting tumor cell migration, increasing ROS generation, and upregulating the expression of Caspase-3 and Caspase-9 in colorectal cancer cells. Overall, the study demonstrated the potential of this DDS for more effective and targeted treatment approaches in colorectal cancer therapy | (Shen et al.,2024) |
| Zr | 2-amino terephthalic acid | 3,4-dihydroxybenzaldehyde (DHBD) and 5-Fluorouracil (5-FU) | UiO-66-NH2-DHBD | pH | intestinal cancer and colorectal cancer cells | chemotherapy | The MOF-based prodrug was engineered to release 3,4-dihydroxybenzaldehyde (DHBD) specifically at the colorectal cancer site. This targeted release was achieved through the pH-sensitive nature of the prodrug, where the C=N bonding of DHBD@MOF was cleaved at the acidic colorectal cancer sites, activating the inactive prodrug efficiently. On the other hand, the drug 5-FU was intended to be released at the intestine. The dual drug delivery system was able to control the release of both DHBD and 5-FU at specific sites by leveraging the pH-responsive properties of the MOF-based prodrug and the drug delivery platform. Overall, this smart dual drug delivery system demonstrated promising potential for targeted drug delivery in cancer therapy. By enabling the selective release of different drugs at specific locations within the gastrointestinal tract, this system could enhance the efficacy of cancer treatment while minimizing side effects and toxicity associated with traditional chemotherapy. | (Binaeian and Rohani, 2024) |
| Al | chitosan (CS) | DOX | Al-MOF/GO | pH | MCF-7. | Cancer treatment | synthesis of chitosan-coated Al-MOF/GO bio-nanohybrid microspheres loaded with doxorubicin (DOX) and 5-fluorouracil (5-Fu) for dual drug delivery in cancer therapy. The study demonstrated high drug encapsulation efficiency, with the total percentage of released 5-Fu and DOX being 63.10% and 54.47%, respectively, under acidic conditions (pH 5.0). In normal conditions (pH 7.4), only 27.69% and 25.10% of 5-Fu and DOX were released. The chitosan-coated microspheres exhibited lower toxicity than the uncoated nanohybrid, indicating enhanced biocompatibility. Additionally, qualitative cell uptake images confirmed the internalization of the chitosan-coated microspheres within cells over 24 hours of incubation, highlighting their potential for pH-sensitive controlled drug delivery in cancer treatment. | (Aghazadeh Asl et al., 2023) |
| Potassium | β-Cyclodextrin (β-CD) | curcumin (CCM) | Glutamine-β-CD-MOF | Glutamine and amino group | MCF-7,  AGS,  NIH/3T3 | Cancer treatment | The investigation into the functionalization of β-Cyclodextrin MOF with Gelatin and Glutamine for Drug Delivery of Curcumin to Cancerous Cells yielded promising results. By integrating L-glutamine into the β-CD-MOF structure, improved cancer cell-specific targeting was achieved, capitalizing on glutamine's crucial role in cancer cell proliferation and energy pathways. The addition of a gelatin coating facilitated controlled drug release in acidic environments, a vital attribute for targeted drug delivery. Impressively, β-CD-MOF@CCM and glutamine-β-CD-MOF@CCM exhibited high drug loading capacities, demonstrating the potential of these frameworks for efficient drug encapsulation. The MTT assay further emphasized the specificity of glutamine-β-CD-MOF in targeting cancerous cells over normal cells, indicating its potential for selective drug delivery. Overall, the functionalized β-CD MOFs displayed controlled drug release and enhanced therapeutic effectiveness, particularly in the realm of cancer therapy, offering a promising avenue for advancing cancer treatment outcomes. | (Sadeh et al., 2024) |

**Table. S1** An overview of reported MOFs used for cancer drug delivery.

**Reference:**

1. Song, M. R., Li, D. Y., Nian, F. Y., Xue, J. P., and Chen, J. J. (2018). Zeolitic imidazolate metal organic framework-8 as an efficient pH-controlled delivery vehicle for zinc phthalocyanine in photodynamic therapy. J. Mater. Sci. 53, 2351–2361. doi: 10.1007/s10853-017-1716-z

2. Kim, K., Lee, S., Jin, E., Palanikumar, L., Lee, J. H., Kim, J. C., et al. (2019). MOF × Biopolymer: Collaborative Combination of Metal-Organic Framework and Biopolymer for Advanced Anticancer Therapy. ACS Appl. Mater. Interfaces 11, 27512–27520. doi: 10.1021/acsami.9b05736

3. Zhu, L., Gui, T., Song, P., Li, W., Wang, J., Hu, C., et al. (2023). Metal-Organic Framework PCN-224 Integrated with Manganese Dioxide, Platinum Nanoparticles, and Glucose Oxidase for Enhanced Cancer Chemodynamic Therapy. ACS Appl. Nano Mater. doi: 10.1021/acsanm.3c00610

4. Dong, J., Ma, K., Ding, J., Pei, Y., and Pei, Z. (2022). pH-responsive Mannose-modified ferrocene Metal-Organic frameworks with rare earth for Tumor-targeted synchronous Chemo/Chemodynamic therapy. Bioorganic Med. Chem. 69. doi: 10.1016/j.bmc.2022.116885

5. Adhikari, C., Das, A., and Chakraborty, A. (2015). Zeolitic Imidazole Framework (ZIF) Nanospheres for Easy Encapsulation and Controlled Release of an Anticancer Drug Doxorubicin under Different External Stimuli: A Way toward Smart Drug Delivery System. Mol. Pharm. 12, 3158–3166. doi: 10.1021/acs.molpharmaceut.5b00043

6. Zhao, X., He, S., Li, B., Liu, B., Shi, Y., Cong, W., et al. (2023b). Xiaoyuan Zhao, # Shipeng He, # Bo Li, Bin Liu, Yejiao Shi, Wei Cong, Fei Gao, Jingjing Li, Fan Wang, Kai Liu, Chunquan Sheng, * Juanjuan Su, * and Hong-Gang Hu *. doi: 10.1021/acs.nanolett.2c04042

7. Binaeian, E., and Rohani, S. (2024). pH-responsive prodrug containing Zr-based MOF and aldehyde-based drug; anionic hydrogel coating as a smart delivery system. Adv. Powder Technol. 35, 104316. doi: 10.1016/j.apt.2023.104316
